# Divergent Brain Network Activity in Asymptomatic *C9orf72* and *SOD1* Variant Carriers Compared With Established Amyotrophic Lateral Sclerosis

**DOI:** 10.1002/hbm.70345

**Published:** 2025-10-03

**Authors:** Michael Trubshaw, Chetan Gohil, Evan Edmond, Malcolm Proudfoot, Katie Yoganathan, Joanne Wuu, Alicia Northall, Oliver Kohl, Charlotte J. Stagg, Anna C. Nobre, Kevin Talbot, Alexander G. Thompson, Michael Benatar, Mark Woolrich, Martin R. Turner

**Affiliations:** ^1^ Oxford Centre for Human Brain Activity, Wellcome Centre for Integrative Neuroimaging University of Oxford Oxford UK; ^2^ Nuffield Department of Clinical Neurosciences University of Oxford Oxford UK; ^3^ Department of Psychiatry University of Oxford Oxford UK; ^4^ Department of Neurology University of Miami Florida USA

**Keywords:** amyotrophic lateral sclerosis, biomarker, C9orf72, genetic, machine learning, magnetoencephalography, motor neuron disease, networks, presymptomatic, SOD1

## Abstract

Understanding the presymptomatic biology in those at high risk of developing amyotrophic lateral sclerosis (ALS) is essential for the development of preventative therapeutic interventions. Approximately 10% of ALS is associated with a *C9orf72* expansion or pathogenic variants in *SOD1*. Magnetoencephalography (MEG), combined with machine learning algorithms, can model brain network dynamics in such at‐risk populations to develop pathogenic biomarkers. Individuals with symptomatic ALS (symALS, *n* = 61), asymptomatic *C9orf72* carriers (aC9, *n* = 16), or pathological *SOD1* carriers (aSOD, *n* = 12), and healthy controls (*n* = 84) underwent resting‐state MEG recordings. Extracted metrics included regional oscillatory power, connectivity, and spectral shape. ‘DyNeMo’ was trained to identify six functional dynamic brain networks. Metrics were compared between groups. A classifier was trained to distinguish asymptomatic gene carriers from controls. Compared to controls, beta frequency power was decreased in both symALS and aC9 groups. The aC9 group showed a marked slowing of frontal oscillatory activity, while the aSOD group showed a marked acceleration. Dynamic network coactivation was dramatically disrupted in aC9, more than in both symALS and aSOD. The classifier accurately distinguished genetically at‐risk groups from controls (receiver‐operator‐characteristic area‐under‐curve 0.89). The cerebral network dynamics of aC9 are markedly different from both aSOD and symALS, supporting the concept of profoundly different upstream pathways in *SOD1* ALS, sparing wider cortical pathology when compared to *C9orf72* ALS. aC9 changes may reflect chronic adaptive changes relating to neurodevelopmental factors or underpin aspects of system vulnerability that define penetrance variability. MEG metrics might provide important biomarkers of prevention therapy efficacy and phenoconversion in at‐risk populations.

## Graphical abstract

*Box A* ‐ dynamic and time‐averaged (static) resting state MEG metrics extracted from four participant groups: symptomatic amyotrophic lateral sclerosis (ALS), asymptomatic *C9orf72* expansion carriers, asymptomatic *SOD1* variant carriers, and healthy controls. *Box B* ‐ summary of MEG‐de rived metric differences associated with each group compared to healthy controls, with some particularly marked changes in the asymptomatic *C9orf72* expansion carriers. *Box C* ‐ dynamic network maps showing marked coactivation of regional networks in asymptomatic *C9orf72* expansion carriers compared to asymptomatic *SOD1* variant carriers.

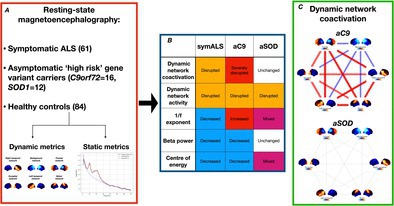

**Summary**

- Resting‐state magnetoencephalography (MEG) can uniquely characterize dynamic and static cortical networks in the pre‐symptomatic phase of neurodegenerative disorders such as amyotrophic lateral sclerosis.
- Asymptomatic *C9orf72* expansion and *SOD1* pathological variant carriers show markedly different cortical neurophysiological profiles, suggesting distinct upstream biological pathways that have common clinical endpoints.
- MEG may help to delineate the compensated and decompensated phases of pre‐symptomatic pathogenesis towards the long‐term aim of establishing outcome measures for prevention trials.

## 1 Introduction

Amyotrophic lateral sclerosis (ALS) is a neurodegenerative condition characterised by disintegration of the motor system and its wider cerebral connectome, resulting in progressive muscle weakness and a median survival of 2–3 years from symptom onset (van Es et al. 2017; Levison et al. 2025; Millul et al. 2005). There is a clinical, genetic, and pathological overlap between symptomatic ALS (symALS) and frontotemporal dementia (FTD) (Majounie et al. 2012; Strong et al. 2017). The causes of symALS are multifactorial, but approximately 10% of cases are associated with either an intronic hexanucleotide repeat expansion in *C9orf72* or pathological variants in *SOD1* (Dharmadasa et al. 2022). These are dominantly inherited with variable penetrance, but typically high (approaching full penetrance) within those presenting to clinic with a known family history (Van Daele et al. 2023). In contrast to *SOD1*, individuals with a *C9orf72* expansion may develop FTD instead of, or alongside symALS (Majounie et al. 2012).

Studies of asymptomatic individuals heterozygous for pathogenic gene variants linked to symALS have created the possibility of revealing preclinical neurobiology. They have provided evidence of changes in cortical excitability and a rise in neurofilament light chain (NfL) levels and chitinases in the months (to a few years) before the emergence of first weakness (Benatar et al. 2018; Gray et al. 2020; Vucic and Kiernan 2008). Neuroimaging evidence of far earlier differences (many years before symptom onset) is detectable in *C9orf72* expansion heterozygotes (Bertrand et al. 2018; van Veenhuijzen et al. 2023; Vucic et al. 2008). The antisense oligonucleotide therapy tofersen has demonstrated disease modification in some *SOD1*‐related symALS cases, in which reduction in NfL preceded clinical benefit by several months (Miller et al. 2022). The awareness of a pre‐symptomatic rise in NfL has formed the basis for the first prevention trial in *SOD1*‐related symALS, in which asymptomatic individuals heterozygous for *SOD1* pathological variants are followed up at regular intervals and randomized to tofersen or placebo at the point when their NfL level rises above a pre‐determined threshold (Benatar, Wuu, Andersen, et al. 2022). Endpoints for such studies are currently limited to clinical outcomes (weakness), supported by electromyography, both markers of well‐established pathology.

The natural history of ALS has been posited to comprise a continuum of stages, running from disease susceptibility to manifest ALS onset, with pre‐manifest and prodromal stages lying between (Benatar et al. 2019). For ALS associated with monogenetic variants, the potential for disease is present from conception. The typical delay in emergence of symptoms for several decades and variable penetrance reflects a complex mix of tolerance through compensatory processes and may rely on genetic co‐factors plus cellular and network functional redundancy. Reaching the goal of early prevention will require identification of biomarkers that reflect the earliest ‘compensated’ or ‘decompensated’ changes (Benatar, Wuu, McHutchison, et al. 2022).

Magnetoencephalography (MEG) is a neuroimaging technique that measures magnetic fields generated primarily by neural activity in the cortex, accurate to the millisecond and millimeter level (Cicmil et al. 2014). Healthy brain function is characterized by functional brain networks with identifiable patterns of oscillatory frequency and topology, which interact dynamically over time, even when a person is at rest (Gohil et al. 2022). Alterations in these network dynamics may reflect compensatory changes that could underpin incomplete penetrance or prodromal decompensatory changes in underlying brain biology.

Static (time‐averaged) network activity in MEG is typically estimated by dividing oscillatory activity into canonical frequency bands (delta, theta, alpha, beta, gamma), calculating oscillatory amplitudes and other metrics averaging over time (Stam 2010). Through this method, it was identified that a reduction in motor cortex beta power (oscillatory amplitude), 1/f slope (increased signal complexity), and increased gamma power and connectivity in the extremes of the frequency range characterize symALS pathology (Dukic et al. 2019, 2024; Govaarts et al. 2022; Proudfoot et al. 2016; Riva et al. 2012; Trubshaw et al. 2024). Some of these metrics, including gamma power and connectivity, increase longitudinally in symALS, but precise interpretations of individual dynamic networks and their interactions are limited using this method alone (Govaarts et al. 2022; Metzger et al. 2024). In the broader context of neurodegeneration, it has been posited that 1/f may represent a function of excitatory:inhibitory balance and is increased (less excitable) in Alzheimer's Disease and Parkinson's disease (Helson et al. 2023; Sun et al. 2020; Trubshaw et al. 2024). True dynamic brain network analysis requires powerful computer processing and therefore has only become practical in the last few years. ‘DyNeMo’ (Dynamic Network Modes) is a machine‐learning algorithm that can identify distinct functional brain networks from MEG data and characterize their intra‐ and inter‐network dynamics to the millisecond timescale (Gohil et al. 2022). The model allows for multiple networks to be active at any one time and outputs their time‐varying activity on a per‐individual basis.

To assess the potential of MEG to deliver biomarkers in the presymptomatic space, this study considered the presence of significantly altered dynamic cortical network changes in asymptomatic individuals heterozygous for genetic variants associated with a high risk of ALS, in relation to both healthy control participants and those with established ALS.

## 2 Materials and Methods

### 2.1 Participants

This was a cross‐sectional study of task‐free MEG. Individuals referred to the Oxford Motor Neuron Disease Care and Research Centre and diagnosed with symALS (*N* = 84) (by consultant neurologists and authors M.R.T., K.T., A.G.T.) were eligible for the study. Genetic testing in the clinic was historically offered to those reporting a dominant family history of either symALS, frontotemporal dementia, or age of onset < 45 years (though this has become a routine offer for all new diagnoses since 2021). First‐degree relatives of individuals known to be heterozygous for an ALS‐related genetic variant were invited to take part in a range of research studies in which they would undergo double‐blinded genetic testing on a research basis, with an independent Data Guardian providing anonymised data grouped by genotype ‘at‐risk’ or not for the analysis. Asymptomatic participant groups used in this study were either heterozygous for a *C9orf72* expansion (aC9, *N* = 16) or one of several accepted pathological variants in *SOD1* (aSOD, *N* = 12). Individuals screening negative were added to a healthy control (HC) group that contained spouses and friends of affected symALS index cases with no significant medical or family history, matching for age and sex (*N* = 84). All patients provided informed consent according to documentation and protocols approved by National Research Ethics Service Committees (14/SC/0083, 17/SC/0277). Additional aSOD group participants, part of the University of Miami ‘*Pre‐fALS*’ study (MB, JW), were added under separate IRB approval from the University of Miami (20101021). MEG data from 36 symALS and 51 HC used in this work have previously been reported in (Trubshaw et al. 2024). The remainder of the symALS, HC, aC9, and aSOD MEG data has not previously been reported.

### 2.2 MEG Acquisition

Participants underwent resting‐state MEG for eight to 10 continuous minutes. Participants were instructed to visually fixate on a cross displayed 120 cm in front of them. Prior to MEG acquisition, participant head shape was recorded using a Polhemus 3D tracking system, relative to three fiducial points on the nasion and preauricular landmarks. The location of five Head Position Indicator (HPI) coils, located on the participants' nasion and bilateral supra‐orbital and posterior auricular regions, was continuously monitored in scanner space. The HPI coil and fiducial locations were digitised using the tracking system (Polhemus, EastTrach 3D) to define the subject‐specific cartesian head co‐ordinate system. Fiducial markers were worn for the MEG only. Participants underwent whole‐brain T1‐weighted MRI (3T Siemens Trio, TE/TR = 4.7/1900 ms, flip angle = 8°, 1 mm3 isotropic) within 1 month for MEG co‐registration. All scans were reviewed contemporaneously for structural abnormalities by a neurologist.

### 2.3 Data Processing

The Oxford Centre for Human Brain Activity (OHBA) Software Library (OSL) v0.6.0 Python package was used for data processing (Quinn et al. 2023). The MaxFilter software v2.2 was used to denoise, detect bad channels, and correct for head movement via the temporal signal space separation algorithm (tSSS) (Taulu et al. 2005). A bandpass filter was applied between 0.5 and 125 Hz; notch filters were applied at 50 and 100 Hz; bad segments were removed using default settings; and the data were resampled to 250 Hz. The MNE signal‐space projection algorithm was used to further denoise biological and environmental artifacts, and data were visually inspected to determine algorithm efficacy (Larson et al. 2024; Uusitalo and Ilmoniemi 1997). Coregistration, forward modeling, and beamforming were carried out with OSL's RHINO tool. Data were co‐registered utilizing each participant's three fiducials, > 100 scalp head shape points, and structural MRI when available, and with the MNI 152 1‐mm standard brain when participants were unable to complete MRI scanning due to intolerability or missed scanning appointment (Grabner et al. 2006). A final bandpass filter between 1 and 80 Hz was applied prior to source localization. Data were beamformed to a regular 8 × 8 × 8 mm^3^ dipole grid, using a data covariance matrix regularized to a rank of 60, with dipole orientations calculated by maximizing each dipole's power (Woolrich et al. 2011).

OSL's RHINO “spatial basis” method was employed for parcellation using a Glasser52 parcellation (Kohl et al. 2023), where each parcel time course was taken to be the first principal component from all voxels that form a parcel (Colclough et al. 2015). Multivariate leakage correction was applied to reduce spatial leakage between parcels (Colclough et al. 2015). Finally, parcel sign flipping was carried out using OSL to align the sign of parcel time courses across subjects.

### 2.4 Static Metrics

The static (time‐averaged) power spectral densities (PSDs) were calculated using Welch's method with a Hann window of 2 s from each *z*‐transformed parcel timecourse (Welch 1967). Power was estimated across six canonical frequency bands: delta (1–4 Hz), theta (4–7 Hz), alpha (7–13 Hz), beta (13–30 Hz), low‐gamma (30–48 Hz), high‐gamma (52–80 Hz). To estimate measures of spectral shape, the FOOOF algorithm v1.0.0 was used to parametrize the PSD between 1 and 70 Hz (Donoghue et al. 2020). FOOOF estimates the full PSD and extracts the aperiodic component (which is the full PSD with peaks in the PSD removed), and the periodic component (i.e., the PSD of the peaks, calculated as the full PSD minus the aperiodic component). The 1/f exponent was estimated by calculating the steepness of the aperiodic component. Settings included peak width limit: 0.5–12.0, maximum number of peaks: ∞, minimum peak height: 0.05, peak threshold: 2.0, aperiodic mode: fixed.

Oscillatory slowing was estimated by calculating the center of energy (CoE). CoE was derived by calculating the frequency for which the summated power below and above that frequency in the de‐FOOOFed PSD were balanced using the method formulated by (Krösche et al. 2023). Previous work has found that CoE is reduced in other neurodegenerative diseases like Alzheimer's and Parkinson's disease and may correlate with levels of protein aggregation (Coomans et al. 2021; Krösche et al. 2023; Stoffers et al. 2007). Oscillatory slowing would be represented by a shift of the CoE to the left (decrease), and an acceleration by a shift to the right (increase). Further explanation of how the 1/f exponent and oscillatory slowing were calculated can be found in Figure S2.

Static (time‐averaged) connectivity is a per parcel‐pair measure estimated by first *z*‐transforming the parcel time courses, bandpass filtering for each of the six canonical frequency bands, and then calculating the pairwise amplitude envelope correlations (AEC) for each of the six bands separately (Colclough et al. 2015). Static parcel connectedness is a per parcel measure that corresponds to how functionally connected a parcel is to the rest of the brain, and was estimated by calculating the mean of the AEC between the parcel and all other parcels.

### 2.5 Dynamic Metrics

OSL‐dynamics v1.2.8 was used to infer all dynamic measures (Gohil et al. 2024). The DyNeMo machine‐learning algorithm was trained on the concatenated data from all subjects, blinded to participant groupings (Gohil et al. 2022). Time‐delay embedding (±7 lags) followed by principal component analysis (to reduce to 100 principal components) were applied to the parcel time courses before training DyNeMo. The full hyperparameters are shown in Table S1. To ensure reproducibility across runs and model settings, the algorithm was trained 30 times for 6, 7, and 8 modes (number of networks the model learns from the data). Results from the three models with the lowest variational free energy (a measure of quality of the fitted model) for each of 6, 7, and 8 modes were used for subsequent analysis (Table S2). The final reported results were from the 6‐mode analysis, chosen to obtain a low number of networks to facilitate interpretation. Note that all reported results were robust to the selection of run and number of modes. This approach has been shown to provide consistently reproducible results (Gohil et al. 2024). Each mode in DyNeMo corresponds to a network, characterized by the spatio‐spectral pattern of oscillatory power and coherence between brain regions that occur when the network is active; as such, we interchangeably refer to the DyNeMo modes as networks.

Dynamic metrics compared between groups included: 1. Dynamic parcel connectedness, which is a per network and per parcel measure of how functionally connected a parcel is to the rest of the brain, estimated by calculating the mean of the network coherence between the parcel and all other parcels, averaged over frequency (similar to static parcel connectedness, but calculated for each network separately); 2. Network coactivation, which is a per network pair measure calculated as the correlation over time between the activity time courses of two networks (note that a network's activity time course corresponds to the network's mode time course from DyNeMo); 3. Network activity strength, which is a per network measure calculated as the average value of a network's activity time course; 4. Network activity variability, which is a per network measure calculated as the standard deviation of a network's activity time course; 5. Network activity pattern concentration, which is a per network measure calculated as the kurtosis of a network's activity time course.

### 2.6 Statistical Analyses

Python v3.8.15, GLMTools v0.2.0, and Scipy v1.10.0 were used for all statistical analyses (Andrew J Quinn 2023; Quinn et al. 2024; SciPy 2025). Each metric of static and dynamic cortical activity was used as the dependent variable in separate General Linear Models (GLMs) to model how each metric varies over subjects using regressors describing the disease/genetic groups alongside confound regressors for age, sex, riluzole administration, and missing structural MRI. To assess the group differences pairwise (symALS‐HC, aC9‐HC, aSOD‐HC, aC9‐aSOD), four separate regressors for each group were included (see Figure S1 for further information on GLM design).

The positive linear relationship between beta power and healthy aging has been well documented in previous research (Schäfer et al. 2014). To explore this relationship and compare between participant groups, a further GLM was constructed using age as the regressor. The GLM was used to assess for differences in the aging trajectories of beta power between groups. In order to better visualize changes in the aging trajectories of beta power (rather than to compare groups statistically), parcels in which there was a highly significant increase in beta power with age (*p* < 0.001) in the healthy cohort were identified. Beta power was then extracted from these parcels in all participants (all selected parcels were sensorimotor). The mean beta power across regions was then calculated for each participant, and linear regression was performed and plotted against age to show the relationship between beta power and age for each participant group separately.

The null hypothesis stated that there were no significant differences in the relevant metrics between groups. Statistical significance was determined using non‐parametric permutation testing (Winkler et al. 2014). Family‐wise error rate correction for multiple comparisons, due to fitting a separate GLM to each parcel and frequency (or mode) where relevant, was accounted for by using the maximum *t*‐statistic method. *T*‐statistics were calculated on the true dataset and then for 5000 random permutations of that data. By taking the maximum *t*‐statistic across all parcels and frequencies in each permutation, a null distribution was created against which the *t*‐statistic from the true dataset was compared. *p* values were calculated by computing the proportion of maximum *t*‐statistics from permutations that were smaller than the true *t*‐statistic. *p* < 0.05 was considered significant. All *p* values are reported after correction for multiple comparisons and reported using the notation (*t*(degrees of freedom), *p* value). Contrast of Parameter Estimates (copes) were estimated to illustrate the size of effect of each contrast (Quinn et al. 2024). Where multiple regions showed significant changes for a single metric, a key region was reported to illustrate the effect (find the full list of significant results in Table S4).

### 2.7 Machine Learning Classifier

Scikit Learn's Random Forest Classifier was used to test whether static (time‐averaged) and dynamic network metrics could be used to correctly identify asymptomatic genetically ‘at‐risk’ individuals from healthy controls (Pedregosa et al. 2011). Features available to the classifier included: static power and spectral shape metrics; dynamic parcel connectedness, network coactivation, network activity strength, network activity variability (network activity standard deviation), and network activity pattern concentration (kurtosis). A subset of healthy control participants was selected to match the age distribution of the asymptomatic participants to address the potential confounding effects of age. A sensitivity analysis including all healthy controls was conducted to ensure that the classifier's performance was not sensitive to the specific group of selected controls. Class weights were accounted for when training the classifier. Supplementary analyses included training the classifier to distinguish aC9‐HC and aSOD‐HC.

A nested cross‐validation approach was used. The data were split into train and test sets using a 5‐fold split, therefore retaining 20% of the dataset as a test set. A random forest classifier was trained on the training data (80%). Hyperparameter optimisation was performed using a randomised search approach with 5‐fold cross‐validation on the training data. Feature selection was optimised by iteratively including model‐derived important features. The model with the highest coefficient of determination (receiver operator characteristic area‐under‐curve (ROC AUC) score) was selected as the optimum model. This model was then tested by using it to predict the group probabilities (HC or genetically at‐risk group member) in the unseen 20% of data.

To ensure generalizability and to mitigate the possibility that model performance might be sensitive to the specific 80%/20% split, the training procedure was repeated 5 times for each unique test split of the 5‐fold split. For each iteration, a new blind classifier was trained on the training split and tested on the unique unseen test split (thereby reducing the risk of overfitting). Unseen predictions from each of the 5 folds were collected and used to plot a ROC curve, a precision‐recall (PR) curve, and a confusion matrix. 95% confidence intervals for the ROC and PR AUCs were calculated using DeLong's method (DeLong et al. 1988).

### 2.8 Data Availability

Requests for data access will be considered on submission to the authors and according to the need to maintain double‐blinding to the genetic status of individual participants. Code for data processing and statistical analyses can be found at: https://github.com/alicianorthall/oxford_als/tree/main/MEG/resting_MEG.

## 3 Results

### 3.1 Demographics

Participant characteristics are in Table 1. Of 61 symALS patients, 52 were of apparently sporadic aetiology and 9 had known pathological genetic variants (5 *C9orf72*, 2 *SOD1*, 1 *DCTN1*, 1 *FIG4*). Of 28 participants in the asymptomatic genetically at‐risk group, there were 16 aC9 and 12 aSOD (including *8 A5V* and 4 *I114T*). None of the participants met Strong Criteria for FTD. ECAS data was not available for the aSOD group, but were instead evaluated using the Addenbrooke's Cognitive Examination group mean score 92.36 (standard deviation 2.57) with all but one participant achieving a normal score (> 88%). Riluzole was an active medication in 52.5% of symALS patients.

**TABLE 1:** Participant characteristics.

	Controls	symALS	aC9	aSOD
*N*	84	61^a^	16	12^c^
Age in years, mean (SD)	57 (16)	63 (11)	48 (14)^b^	47 (12)^b^
Male	52%	69%	31%	8%
ALSFRS‐R, mean (SD)	—	38.0 (6.1)	—	—
Years since onset, mean (SD)	—	2.3 (1.5)	—	—
Limb onset	—	80%	—	—
Edinburgh cognitive assessment score (ECAS), mean	120.4 (9.2)^b^	112.9 (9.5)	120.1 (8.7)^b^	Unavailable^d^
Riluzole (%)	0	52.4	0	0

^a^ Including 5 C9orf72, 2 SOD1, 1 DCTN1, 1 FIG4.

^b^ *p* < 0.05 compared to ALS.

^c^ Including 8 A5V and 4 I114T.

^d^ Addenbrooke's cognitive examination score was available (aSOD 92.36% (2.57)). Scoring 0–100, > 88 is normal.

There were no structural abnormalities out of keeping with ALS noted on participant MRI scans. For 10 participants (2 HC, 4 symALS, 3 aC9, 1 aSOD), MRI was not possible. In these cases, the MNI 152 standard brain was used for MEG co‐registration.

A full table of parcellated Glasser52 brain regions and all significant results can be found in the Tables S3 and S4, respectively (Kohl et al. 2023).

### 3.2 Static Network Measures

#### 3.2.1 The Power Profile

*Compared to controls, beta power was reduced, and the normal increase in beta power with age was disrupted in both the symALS and aC9 groups but not aSOD*.

**FIGURE 1:**
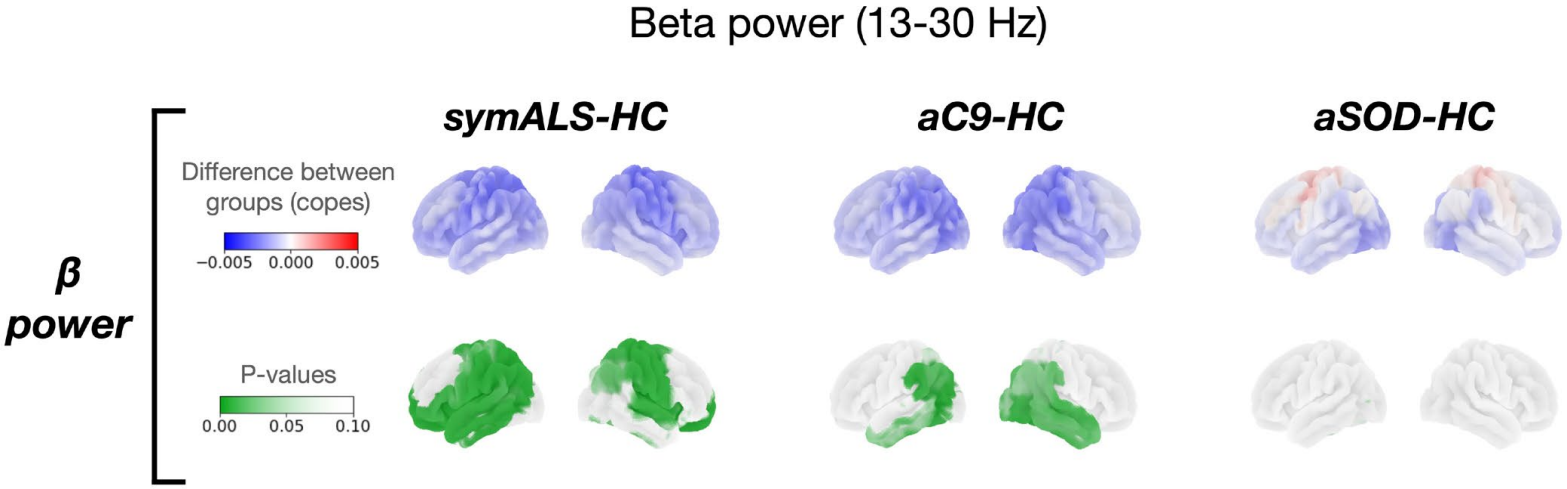
Beta power. Beta power in each disease group (symALS (amyotrophic lateral sclerosis), aC9 (asymptomatic *C9orf72* expansion heterozygous), aSOD (asymptomatic *SOD1* variant heterozygous)) compared to healthy controls (HC). The top row (copes) represents the regional difference in beta power between groups (blue is a negative and red a positive difference). The bottom row (*p* values) represents the regional significance with maximum *t*‐statistic correction for multiple comparisons across regions and frequency bands, with regions of *p* < 0.1 shown in green. symALS was characterised by reduced beta power in temporal, frontal and motor regions. aC9 similarly showed decreased beta power in occipital and temporal regions, but also showed increased frontal theta, and increased alpha in motor and occipital regions aSOD showed no significant changes in beta power.

Compared to HC, there was reduced beta frequency power in the frontal (right polar frontal: (*t*(159) = −2.610, *p* = 0.013), left polar frontal: (*t*(159) = −3.556, *p* < 0.001)) and sensorimotor areas of those with symALS (right cingulate motor: (*t*(159) = −2.610, *p* = 0.013), left inferior motor: (*t*(159) = −3.128, *p* = 0.001)), and the temporo‐occipital regions of the aC9 group (left temporal: (*t*(159) = −3.082, *p* = 0.001), left occipital (*t*(159) = −2.749, *p* = 0.006)) (Figure 1). In contrast to aC9, aSOD showed no significant changes in beta power (*p* > 0.1). Results from the remaining frequency bands can be found in the Figure S3A and Table S4.

Beta power in sensorimotor brain regions increased with age in HC (*t*(159) = 4.954, *p* = 0.001) and a more limited number of regions in aC9 (*t*(159) = 3.285, *p* = 0.029) but not in symALS or aSOD (Figure S3Bi). This relationship between age and beta power was visualized further by plotting mean beta power over sensorimotor regions in Figure S3C and performing linear regression for each group separately. This confirmed a significant increase in beta power across all selected regions in HC (*r* = 0.557, *p* < 0.001), with a different relationship seen in both asymptomatic genetically at‐risk groups that intersects the symALS trajectory at ~55 years old (Figure S3C). The rate of beta power increase with age was significantly lower in symALS and aSOD when compared to HC in motor regions ((*t*(159) = −4.016, *p* = 0.002) and (*t*(159) = −4.082, *p* = 0.005) respectively) (Figure 3Bii).

#### 3.2.2 Spectral Shape

*Compared to controls, the 1/f exponent was reduced in symALS (and aSOD frontal regions) but increased in aC9 (and aSOD occipital regions). There was marked oscillatory slowing (reduced CoE) in symALS and aC9 but oscillatory acceleration (increased CoE) in aSOD (frontal regions)*.

An explanation of spectral shape metrics can be found in Figure S2.

**FIGURE 2:**
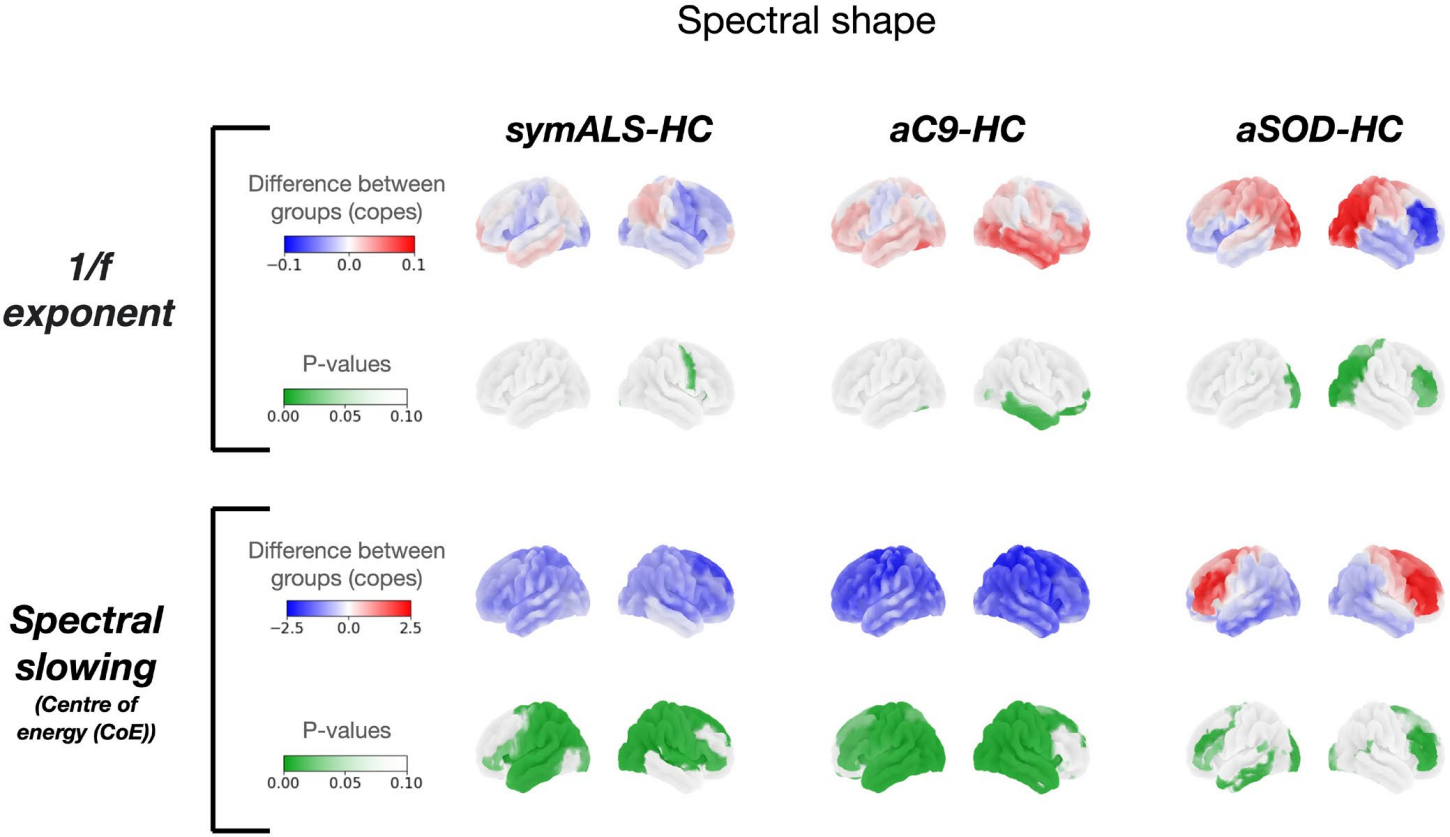
Spectral shape. The top and third row (copes) represent the regional difference in 1/f exponent and centre of energy (CoE) respectively between groups (blue is a negative and red a positive difference). The bottom row (*p* values) represents the regional significance with maximum *t*‐statistic correction for multiple comparisons across regions and frequency bands, with regions of *p* < 0.1 shown in green. The 1/f exponent was reduced in amyotrophic lateral sclerosis (symALS) compared to controls (HC) however asymptomatic *C9orf72* expansion heterozygotes (aC9) showed an increase in fronto‐temporal 1/f whilst asymptomatic *SOD1* variant heterozygotes (aSOD) showed an increase in occipital 1/f and a decrease in frontal regions. The centre of energy (CoE) was reduced in symALS and aC9 when compared to controls. aSOD showed an increase in frontal and a decrease in occipital CoE compared to controls.

Compared to HC, the exponent of the 1/f component of the power spectrum (averaged over time for each subject) was reduced in symALS (right motor: (*t*(159) = −1.896, *p* = 0.014)), but increased in aC9 (right temporal: (*t*(159) = 2.054, *p* = 0.016), right frontal: (*t*(159) = 1.980, *p* = 0.030)) (Figure 2 top). aSOD showed a mixed picture of increased 1/f in the occipital regions (right visual: (*t*(159) = 2.628, *p* = 0.002), left visual: (*t*(159) = 2.298, *p* = 0.004)) and decreased 1/f in frontal regions (right inferior frontal: (*t*(159) = −2.151, *p* = 0.009)) (Figure 2 top row).

Centring around occipital regions, there was widespread oscillatory slowing (reduced CoE) in symALS (right: (*t*(159) = −2.791, *p* = 0.001)), left: (*t*(159) = −3.073, *p* = 0.001), aC9 (right: (*t*(159) = −3.637, *p* = 0.001)), left: (*t*(159) = −4.096, *p* = 0.001) and aSOD (left: (*t*(159) = −2.031, *p* = 0.021)) (Figure 2 bottom). aSOD also showed oscillatory acceleration (increased CoE) in frontal regions; however, (left dorsolateral prefrontal cortex (DLPFC): (*t*(159) = 2.399, *p* = 0.008)) (Figure 2 bottom right). Comparing aC9 and aSOD directly revealed oscillatory slowing in aC9 (significantly reduced CoE) in occipital (right: (*t*(159) = −3.909, *p* = 0.020)) and motor regions (right: (*t*(159) = −3.153, *p* = 0.001)), left: (*t*(159) = −3.489, *p* = 0.003)) compared to aSOD.

#### 3.2.3 Static Connectedness

*Compared to controls*, *symALS showed increased static parcel connectedness in high (gamma, high‐gamma) and low (delta) frequency bands*.

Increases in static parcel connectedness were observed in frontotemporal, occipital, and motor regions in symALS in the delta band (left temporal: (*t*(159) = 2.643, *p* = 0.049), right parietal: (*t*(159) = 2.741, *p* = 0.035)), gamma band (right visual: (*t*(159) = 3.250, *p* = 0.005), left temporal: (*t*(159) = 3.439, *p* = 0.003)), and high‐gamma band (left visual: (*t*(159) = 2.915, *p* = 0.0019), right temporal (*t*(159) = 2.703, *p* = 0.042), right cingulate motor: (*t*(159) = 2.826, *p* = 0.027)) (Figure S4). There were no significant differences in parcel connectedness between asymptomatic genetic at‐risk groups and HC.

A full table of static results can be found in Table S4.

### 3.3 Dynamic Network Measures

#### 3.3.1 Network Descriptions

Six functional networks were extracted from the DyNeMo model with the lowest variational free energy (a metric summarising how well the model represents the data) across 90 runs: right temporal, background, frontal, occipital, left temporal, and motor networks. Each network was characterised by its unique topographical power profile, connectivity, and whole‐brain PSD (Figure S5A). The background mode contains information unexplained by the other functional networks and therefore reflects static ‘background’ activity to an extent. Its activity, however, also fluctuates over time (Gohil et al. 2022). Figure S5B shows an example timecourse derived from the DyNeMo model to illustrate how mode activity fluctuates over time.

#### 3.3.2 Network Summary Statistics

*Compared to controls, all three patient groups showed significant changes in occipital network activity: symALS temporal and frontal network changes*.

symALS, aC9, and aSOD all showed increased occipital network variability ((*t*(159) = 1.921, *p* = 0.009)), (*t*(159) = 2.671, *p* = 0.001), (*t*(159) = 2.575, *p* = 0.004) respectively) compared to controls. aC9 and aSOD showed increased occipital network activity strength ((*t*(159) = 2.045, *p* = 0.001), (*t*(159) = 2.355, *p* = 0.001) respectively) compared to HC (Figure S6—column 4). symALS showed an increase in frontal network activity variability (*t*(159) = 3.02, *p* < 0.001) (Figure S6—column 3). Motor network activity variability was increased in aSOD (*t*(159) = 1.879, *p* = 0.021). Left temporal network activity strength was reduced in symALS and aC9 ((*t*(159) = −1.029, *p* = 0.012), (*t*(159) = −1.903, *p* = 0.005) respectively) Figure S6—column 5). Background network activity strength was increased in symALS and aC9 ((*t*(159) = 1.992, *p* < 0.001), (*t*(159) = 1.520, *p* = 0.021) respectively), and variability increased in symALS (*t*(159) = 2.181, *p* = 0.003)) (Figure S6—column 2).

During analysis it was noted that the addition of riluzole as a regressor to the model had a disproportionate effect on network summary statistics results. A supplementary analysis was therefore conducted on the most robust finding from the summary statistics: increased frontal network activity variability in symALS. The effect of riluzole on this metric was evaluated in the symALS group only, accounting for age, sex, missing structural and ALSFRS, thereby mitigating the effect of disease burden. Riluzole significantly reduced frontal network activity variability (*t*(49) = 1.947, *p* = 0.045), suggesting that riluzole at least partially corrected abnormally high frontal network variability in symALS.

#### 3.3.3 Network Coactivations

*Compared to controls*, *both symALS and aC9 showed disrupted network co‐activations*.

**FIGURE 3:**
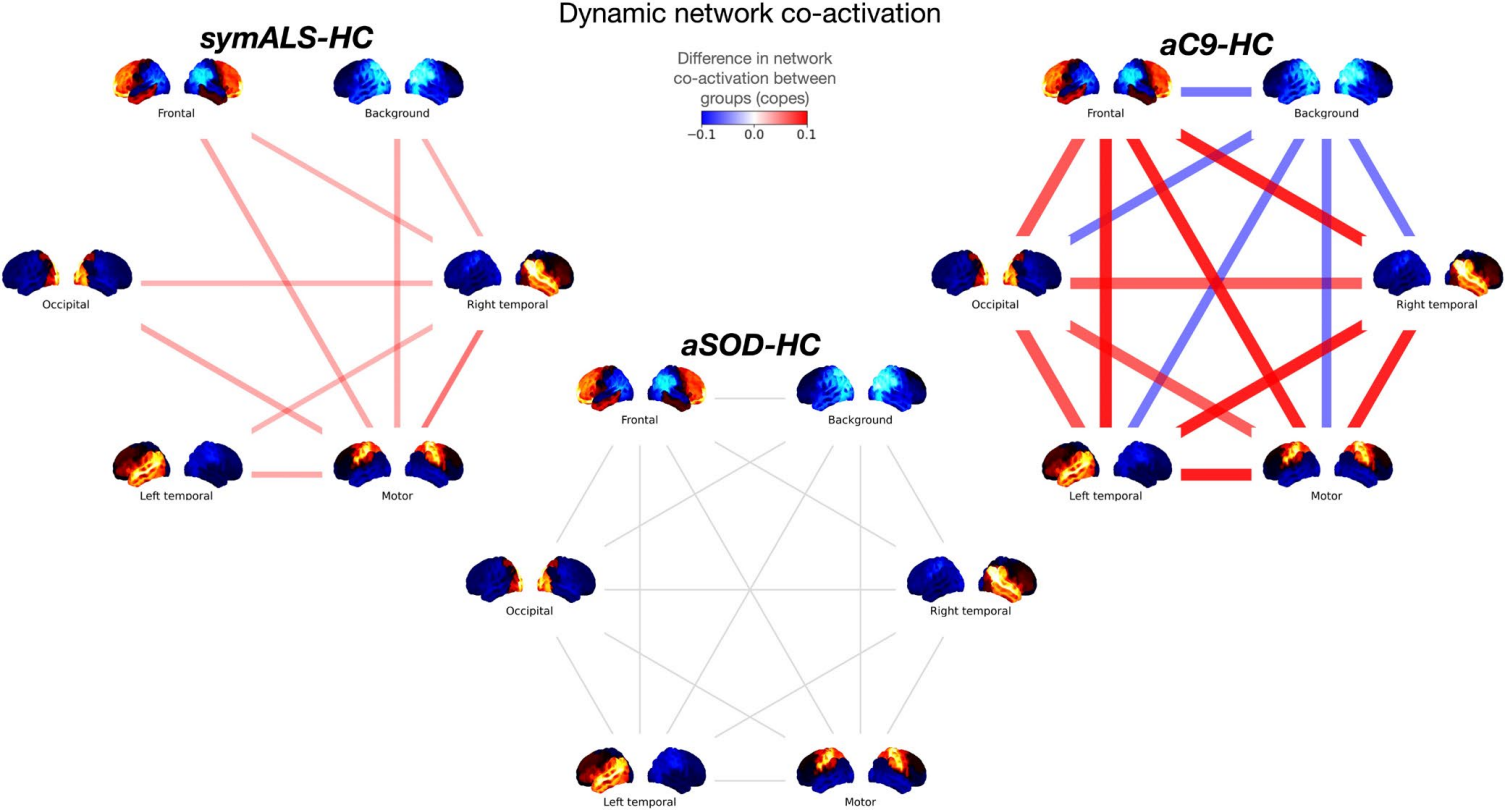
DyNeMo network co‐activations. The difference in network co‐activation in each disease group compared to healthy controls (HC). Red connections represent an increase in co‐activation whilst blue connections represent a decrease in co‐activation. Thicker and more opaque lines represent larger differences in co‐activation between groups. Only connections with *p* < 0.05 were plotted. The size of the difference between the groups (copes) is represented by the width and opacity of the connection. symALS showed a significant increase in co‐activation of all other networks with the right temporal and motor networks. aC9 showed large and highly significant increases in the co‐activation of all networks with the right temporal, left temporal, motor, frontal and occipital networks when compared to controls. Only the background network showed a significant decrease in co‐activation compared to controls.

The right temporal and motor networks showed increased network coactivation in symALS (*t*(159) = 1.316, *p* = 0.050 and *t*(159) = 1.813, *p* = 0.008 respectively) implying that the activity of these networks was more highly correlated with all other networks compared to HC (Figure 3—left). The left temporal network was also more highly coactivated in symALS compared to controls although this did not reach statistical significance after multiple comparison correction (*t*(159) = 1.240, *p* = 0.078). In aC9 there were markedly increased network coactivation of the right temporal (*t*(159) = 1.940, *p* = 0.011), occipital (*t*(159) = 1.819, *p* = 0.016), left temporal (*t*(159) = 1.960, *p* = 0.010), and motor networks (*t*(159) = 1.679, *p* = 0.029). In contrast, the background network was negatively coactivated (*t*(159) = −1.415, *p* = 0.045) (Figure 3—right). There were no significant differences in aSOD‐HC (Figure 3—bottom) or aC9‐aSOD contrasts.

#### 3.3.4 Dynamic Connectedness

*Intra‐network parcel connectedness was increased in symALS and aSOD in all dynamic networks, whereas in aC9 only temporal, frontal, and occipital intra‐network parcel connectedness was increased*.

Increased parcel (intra‐network) connectedness in symALS was observed across all dynamic networks in frontal, occipital, and temporal regions compared to HC. Increased parcel connectedness was also observed in aC9 but limited to right temporal, left inferior frontal, and occipital networks. aSOD also showed cross‐network increases in parcel connectedness, but these were limited to occipital and superior motor regions. There were no significant differences in parcel connectedness between aC9 and aSOD. See Table S5 and Figure S7 for full results.

### 3.4 Random Forest Classifier

*The genetically at‐risk groups could be distinguished from healthy controls using resting state MEG data with a sensitivity of 71.4% and specificity of 82.5%*.

**FIGURE 4:**
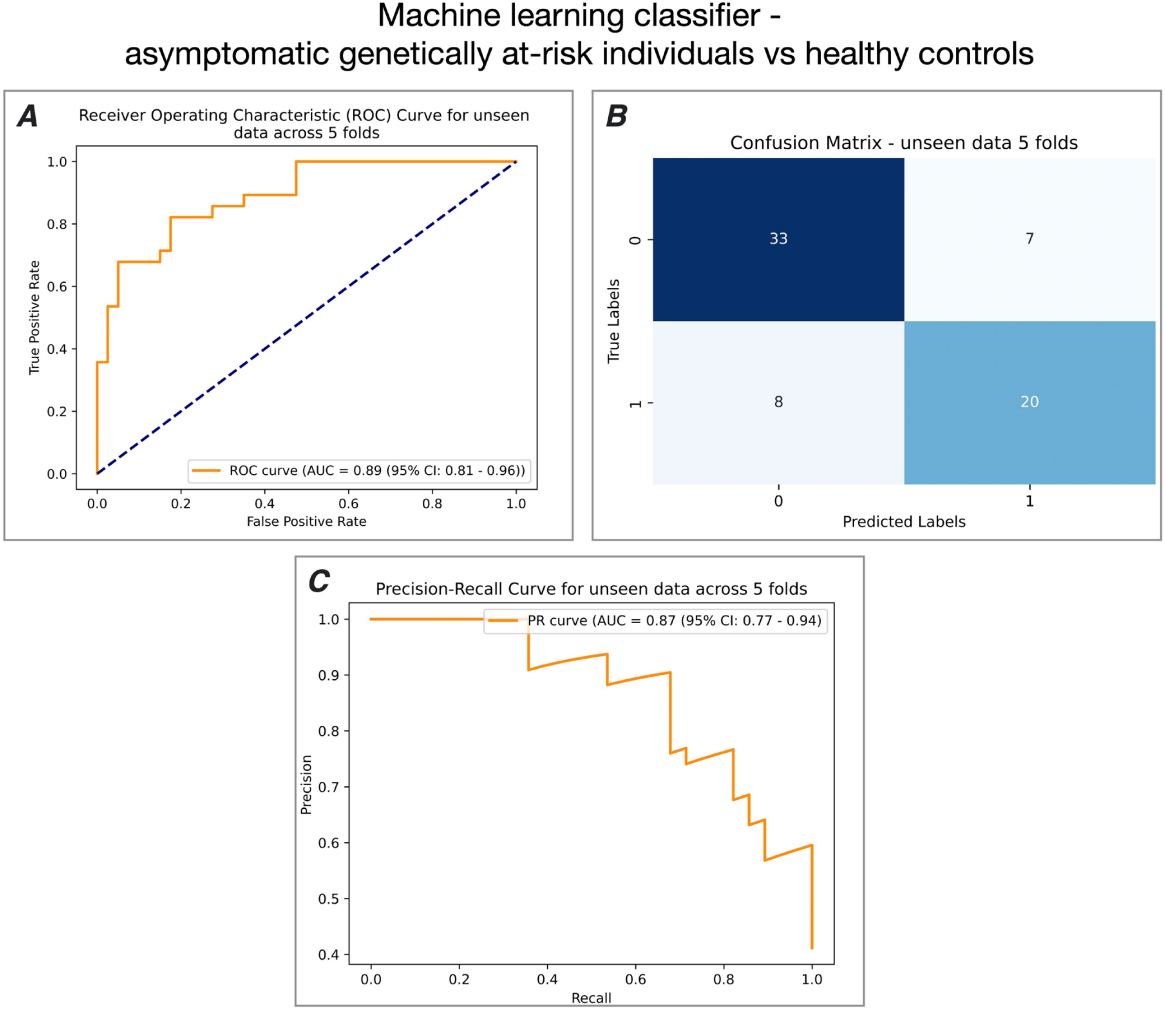
Random Forest Classifier performance predicting asymptomatic genetically at‐risk individuals (aC9 and aSOD) from matched HC. (A) ROC curve for unseen predictions across 5 folds. The Random Forest Classifier achieved a ROC AUC of 0.89. (B) Confusion matrix. Shows classifier performance and indicates a sensitivity of 71.4% and specificity of 82.5% for detecting asymptomatic carrier status in unseen data. (C) Precision‐recall (PR) curve. Shows a classifier performance PR AUC of 0.87.

Using nested cross‐validation, a Random Forest Classifier was trained to detect asymptomatic genetically at‐risk status (as a single group *N* = 28 (16 aC9, 12 aSOD)) from age‐matched HC (*N* = 40). There was no significant difference in the ages of the groups (HC = 48.05 years, genetically at‐risk = 47.71 years, *t*(59.53) = −0.099, *p* (uncorrected) = 0.92). Predictions across 5 folds of unseen data (20% (13 or 14) participants were retained as unseen in each fold) produced a ROC AUC of 0.89 (95% CI: 0.81–0.96) and a precision‐recall (PR) curve AUC of 0.87 (95% CI: 0.77–0.94) (Figure 4A,C respectively). The confusion matrix is shown in Figure 4B). To prove that the classifier was not overfitted to the specific subgroup of age‐matched HC, a supplementary analysis trained a classifier to distinguish between asymptomatic genetically at‐risk status (as a single group *n* = 28 (aC9 *n* = 16, aSOD *n* = 12)) from all healthy controls (*n* = 84) and achieved a ROC AUC of 0.89 (95% CI: 0.82–0.94) and a PR AUC of 0.68 (95% CI: 0.58–0.76).

A supplementary analysis trained a further two Random Forest Classifiers to predict aC9 from HC (ROC AUC 0.83 (95% CI: 0.72–0.92) (Figure S8)), and aSOD from HC (ROC AUC 0.82 (95% CI: 0.70–0.91) (Figure S9)). Performance of these two classifiers was likely reduced compared to the asymptomatic genetically at‐risk classifier because of the smaller sample size on which the Random Forest Classifier was trained (16 aC9 and 12 aSOD respectively, compared to 28 combined).

## 4 Discussion

At a group level, asymptomatic individuals at high genetic risk of ALS and also FTD due to a pathogenic expansion in *C9orf72* showed significant MEG‐derived cerebral neurophysiological differences compared to healthy controls. While some of these differences overlapped with changes seen in a group of people with ALS, there were striking distinctions between the two genetically at‐risk groups hinting at distinct cortical neurophysiology. The aC9 group showed very marked dynamic network differences involving a global network hyper‐coactivation compared to ALS, in marked contrast to the minimal differences seen in aSOD (although genetic heterogeneity within this cohort must be noted). Additionally, an increase in the 1/f exponent in aC9, reflecting changes in network complexity that were noted in a previous study of ALS, may more specifically reflect changes in cortical excitatory‐inhibitory balance (Gao et al. 2017; Gerster et al. 2022; Trubshaw et al. 2024).

Studies using MRI and PET have reported differences in aC9 brain structure and function including cortical, subcortical, and cervical cord atrophy, reduced white matter tract integrity, hypometabolism in frontotemporal and deep structures, and reduced neurite density (Barry et al. 2022; De Vocht et al. 2020; Floeter et al. 2016; Le Blanc et al. 2020; Lee et al. 2017; Menke et al. 2016; Papma et al. 2017; Querin et al. 2019; van Veenhuijzen et al. 2023; Wen et al. 2019). A recent EEG study in an asymptomatic group of *C9orf72* expansion carriers found significantly altered regional cortical activity during a Go/NoGo task (Dukic et al. 2025). Findings in other brain imaging studies of asymptomatic *SOD1* variant heterozygotes, however, have been notably distinct, with a similar relative sparing of cerebral motor involvement despite similar levels of disability and clinical corticospinal tract signs, and a presumption of potentially more cord‐based pathology (Agosta et al. 2018; Stanton et al. 2009; Turner et al. 2005). Although often *clinically* indistinguishable in established symptomatic disease, *SOD1* ALS is notable in lacking the characteristic neuronal and glial cytoplasmic inclusions of TDP‐43 that are found in 97% of all other cases of ALS (and 50% of FTD), including those mediated by *C9orf72* (Mackenzie et al. 2007). The incidence of overt cognitive impairment in *SOD1* ALS is also markedly lower (Wicks et al. 2009). Therefore, the striking contrast in the MEG dynamic network coactivation changes in *C9orf72* and *SOD1* carrier groups in this study (with the caveat of variable genotypes comprising the latter) strengthens the hypothesis that the upstream molecular pathways differ profoundly enough to result in a divergence of presymptomatic cortical neurophysiological changes, some of which may be adaptive.

Corroborating previous studies, the static network analysis in the symALS cases is characterized by decreased motor cortical beta power, 1/f exponent, and increased high‐gamma power in symALS when compared to HC (Dukic et al. 2019, 2024; Govaarts et al. 2022; Proudfoot et al. 2016; Proudfoot, Van Ede, et al. 2018; Riva et al. 2012; Sevelsted Stærmose et al. 2022; Trubshaw et al. 2024). The apparent shift from increased 1/f exponent in asymptomatic groups to reduced 1/f in symALS supports suggestions from TMS studies that cortical hyperexcitability might manifest at the point of phenoconversion (Geevasinga et al. 2015; Pradhan and Bellingham 2021; Vucic et al. 2008). The reduced beta power seen across the disease groups may reflect changes in GABAergic interneurons, a hypothesis with a range of supporting evidence (reviewed in Turner and Kiernan 2012) and which has been linked independently to the degree of gamma band activation in symptomatic ALS patients during a motor task (Trubshaw et al. 2025). Future work should seek to establish the precise mechanism behind beta disruption in symptomatic and asymptomatic ALS, through focus on beta bursts (West et al. 2023; Yoganathan et al. 2025). This study also builds on previously observed dynamic network changes in ALS (Metzger et al. 2024; Notturno et al. 2023; Polverino et al. 2022). Both delta and gamma oscillations play a pivotal role in long‐range region‐region communication (Tóth et al. 2012). The whole‐brain delta and gamma connectivity increases observed in ALS raise the question of whether motor dysfunction in ALS results from wider disintegration of non‐motor functional networks (Stanziano et al. 2024). These results support the overwhelmingly accepted view that both motor and non‐motor networks are disrupted in ALS.

This work adds to previous dynamic EEG microstate work (Metzger et al. 2024) by providing a spatio‐spectral neurophysiological description of symALS and its risk groups. Eliminating the assumption of network mutual exclusivity, DyNeMo, unlike microstates, allows the identification and analysis of the dynamics of multiple networks simultaneously while taking into account both the regional and spectral profile of brain activity (Gohil et al. 2022). The frontal network showed higher variance of activity in ALS and provides evidence for the theory that more disordered within‐network communication may underpin the more subtle cognitive changes often present in ALS cases without dementia (Finsel et al. 2023). The left temporal network showed reduced activity in symALS, but the background network (which represents unexplained activity by other functional networks) was hyperactive. Hyperactivity has previously been observed in EEG (McMackin et al. 2020, 2021) and MRI (Ghaderi et al. 2023). Plasticity, even in the degenerating brain, may underpin background hyperactivity, disruption to occipital network activity, coactivation of the temporal and motor networks, and increased intra‐network connectedness (i.e., parcel connectedness) which were seen consistently across all networks in the present study. Increased connectivity has been a consistent feature among MEG studies in ALS and FTD (Dukic et al. 2019; Govaarts et al. 2021). Occipital changes have been reported in fMRI (Stanziano et al. 2024) and MEG (Proudfoot, Colclough, et al. 2018). Changes in occipital activity in ALS are perhaps unexpected as traditionally these areas were thought to be spared. Further in‐depth specific investigation is required to characterize the meaning of these findings further.

It must be noted that the symptomatic group in this study was essentially ALS‐only (with no clinical FTD) and nearly all *C9orf72* expansion *negative* cases. Therefore, the apparently more modest changes in the dynamic networks of symptomatic ALS cases compared to the aC9 group must be seen in the context of the aC9 group containing individuals at risk of FTD as well as ALS, with the symptomatic group, in contrast, being motor (ALS)‐predominant. It will be necessary to compare (through multi‐centre collaboration) an asymptomatic group with a larger group of both symptomatic ALS and symptomatic FTD C9orf72 expansion heterozygotes, which might also help to untangle the contribution of any neurodevelopmental differences associated with this genotype.

A Random Forest Classifier showed that both static and dynamic MEG data in the resting state robustly distinguished asymptomatic genetically at‐risk individuals from healthy controls. The nested cross‐validation approach we used provided generalizability of this model to new data and therefore testable on future patients, given the limitation of our small group size. The high ROC AUC (0.89), sensitivity (71.4%) and specificity (82.5%) of the classifier are promising and bring aspirations of population‐level presymptomatic screening for ALS marginally closer. Large‐scale longitudinal work is required to advance this field; a screening test's specificity must approach 100%. 10 of our participants did not have an MRI available and therefore MEG data was co‐registered using the MNI‐152 standard brain for these participants. Alignment errors may have occurred in this process; however, we mitigated these by including a covariate in the GLM. MEG shows superior temporal resolution when compared to MRI; however, it provides little structural information. Future studies might seek to combine MRI and MEG metrics to capitalize on the strengths of both modalities. Although riluzole administration was controlled for in the GLMs, a full medication list was not available for every asymptomatic genetically at‐risk participant, introducing potential confounding. Although correction for differences in age and sex was made in the GLMs, it should be noted that the genetically at‐risk groups had different demographic profiles compared to the HC group. The link between 1/f and the excitability remains contentious, although studies have shown expected shifts in 1/f as a result of administration of benzodiazepines and general anesthetic (Akbarian et al. 2023; Gao et al. 2017). The effect of age on beta power was assumed to be linear; further work is planned to assess this assumption. The penetrance of both the *C9orf72* hexanucleotide repeat expansion and the *SOD1* gene variants studied here cannot be assumed to be complete. Group‐level changes described here and in many other studies across modalities may therefore reflect a mixture of complex neurodevelopmental differences, chronic compensatory and potentially mitigating responses, as well as some perhaps more indicative of early pathological change. We believe this is an important shift in perspective that needs to be considered in much more detail, especially as preventative interventions begin to be contemplated. Unraveling the issue of incomplete penetrance will require much larger (genetically homogeneous) groups of asymptomatic individuals across a range of ages. This will undoubtedly be informed in parallel by an expected growth in the discovery of genetic modifiers of risk and other presymptomatic factors linked to penetrance.

The aspiration to begin prevention trials cannot be based on an outcome measure of symptomatic phenoconversion from longitudinal studies necessarily lasting decades. Outcome measures must ultimately be clinically meaningful or validated surrogates. Sensitive, non‐invasive biomarkers of cortical neurophysiology showing significant deviation from ‘healthy’ ageing might be identifiable from larger cross‐sectional studies, using MEG or its inevitable more portable derivatives. These measures offer tangible hope for defining a critical phase of decompensation in those already at genetic risk, which might form a trial entry point for preventative interventions.

## Conflicts of Interest

Board member is co‐author: Mark Woolrich is a member of HBM Editorial Board and co‐author of this article.

## Supporting information


**Figure S1:** General linear model design—group comparison. Design matrix used to predict network metrics. The first regressor (HC) models the mean value of the network metric across healthy controls. The second regressor models the mean value of the network metric across symALS patients. The third and fourth (aC9 and aSOD) model the mean values for each asymptomatic carrier group respectively. The remaining regressors are included to model known sources of variability (age, sex, missing structural) across participants. This has the effect of minimising the impact of these confounds on the group means. The confound regressors are calculated by *z*‐transforming the values for age, sex (1 = female or 2 = male) and missing structural (1 = not missing, 2 = missing) across participants.
**Figure S2:** Explanation of spectral shape metrics. The top graph shows an example annotated power spectrum density curve (PSD). The red line represents the FOOOF full model fit. The blue dashed line represents the aperiodic component. The 1/f exponent is derived by taking the steepness of the slope of the aperiodic fit. The bottom graph shows the same PSD after the aperiodic fit has been subtracted from the full model fit, and therefore represents an estimation of the periodic component. The CoE is derived by calculating the frequency at which the sum of the power below = the sum of the power above (represented by the red line). Spectral slowing would be represented by a shift of the CoE to the left.
**Figure S3:** (A) The power profile. Oscillatory power in each disease group compared to healthy controls. symALS was characterised by reduced beta and increased high‐gamma power in motor regions. aC9 similarly showed decreased beta power in occipital and temporal regions, but also showed increased frontal theta, and increased alpha in motor and occipital regions. aSOD showed an increase in frontal gamma, but a decrease in occipital gamma and high‐gamma, and a decrease in frontal theta. (B) Beta power age trajectory. (i) Shows the effect of age on cortical beta power. Most brain regions in HC showed a significant increase in beta power with age. Sensorimotor regions showed a significant increase of beta power with age in aC9. (ii) Shows the parcel‐wise difference in age trajectories between disease groups. Both symALS and aSOD showed a significantly reduced effect of age in sensorimotor regions compared to HC. (C) Beta power and age. Linear regression to show the effect of age in each disease group. Mean beta power from the 29 parcels which had a *p* < 0.001 effect of age in the HC cohort were extracted from each participant and plotted here. Each data point represents the mean beta power across 29 parcels in each participant. The regression lines were calculated by performing linear regression on the mean beta power across the 29 regions. *p* values are displayed after Bonferroni correction for multiple comparisons. Beta power generally increased with age in the HC cohort (*r* = 0.557, *p* < 0.001). Deviation from healthy (a reduction in beta power) occurred between 50 and 60 years of age in symALS. aSOD showed higher, whilst aC9 showed lower beta power when compared to controls prior to reaching their 50s. By the time they reached their 60s, both genetically at‐risk groups aligned with the symALS group, having come from different ‘sides’ of the healthy beta range.
**Figure S4:** Static parcel connectedness profile. Per parcel functional connectivity between each parcel and the rest of the brain was increased in fronto‐temporal regions in delta, gamma and high‐gamma frequency bands in symALS compared to controls. aC9 and aSOD showed no significant changes in the delta band compared to controls. aC9 showed an increase in static parcel connectedness in right fronto‐temporal regions in the theta band. aSOD showed a small area of increase in the right occipital parcel compared to controls in the high‐ gamma band.
**Figure S5:** (A) DyNeMo network descriptions. The first two rows show the power maps across participants of the six networks extracted from DyNeMo and the associated connectivity maps (strongest 5% of connections) respectively. The third row shows the associated network PSDs with the black dotted line representing the mean PSD across networks and the coloured line representing the network‐ specific PSD. (B) DyNeMo example time course. Shows an example of one participant's DyNeMo time course. The proportion of total brain activity explained by each mode (1–6) varies over time. This proportion is represented by the thickness of each colour at each time point.
**Figure S6:** DyNeMo network summary statistics. The first row shows the power maps across participants of networks extracted from DyNeMo. The last three rows show group‐comparisons from a General Linear Model comparing network activity strength, network activity variability (standard deviation) and network activity pattern concentration (kurtosis) respectively for each network. Maximum *t*‐ statistic correction for multiple comparisons was applied across networks. Compared to controls (HC), people with amyotrophic lateral sclerosis (symALS) showed decreased network activity strength in the left temporal network but increased network activity strength in the background network. The network activity variability was increased in symALS in the frontal, occipital and background networks. Asymptomatic *C9orf72* carriers (aC9) showed an increase in occipital and background network activity strength and a decrease in left temporal network activity strength. aC9 occipital network activity variability was increased. Asymptomatic *SOD1* carriers (aSOD) showed increased occipital network activity strength and decreased right temporal network activity strength. The occipital and motor networks both had a higher activity variability in aSOD.
**Figure S7:** DyNeMo network connectivity comparisons. Shows GLM results from group‐level comparisons of global connectivity in each network. symALS showed increased dynamic (intra‐network) parcel connectedness in all networks in all regions other than the motor cortex. aC9 and aSOD showed smaller areas of significant increase in all networks in frontal, temporal and occipital regions.
**Figure S8:** Random forest classifier performance predicting aC9 from matched HC. (A) ROC curve for unseen predictions across 5 folds. The random forest classifier achieved a ROC AUC of 0.83. (B) Confusion matrix. Shows classifier performance. (C) Precision‐recall (PR) curve. Shows a classifier performance PR AUC of 0.61.
**Figure S9:** Random forest classifier performance predicting aSOD from matched HC. (A) ROC curve for unseen predictions across 5 folds. The random forest classifier achieved a ROC AUC of 0.82. (B) Confusion matrix. Shows classifier performance. (C) Precision‐recall (PR) curve. Shows a classifier performance PR AUC of 0.54.
**Table S1:** DyNeMo hyperparameter settings.
**Table S2:** DyNeMo free energy scores. 30 models were trained for each number of modes. The run with the lowest free energy from each mode selection is displayed here.
**Table S3:** Glasser52 parcellation co‐ordinates. Parcel names and MNE‐coordinates of parcel centers of the Glasser52 Parcellation.

**Table S4:** Full table of static results. Summarizes statistical analysis and results for each measure of cortical activity in the static analysis.
**Table S5:** Full table of dynamic results. Summarizes statistical analysis and results for each measure of cortical activity in the dynamic analysis.

## Data Availability

The data that support the findings of this study are available on request from the corresponding author. The data are not publicly available due to privacy or ethical restrictions.
