# Supplementary material for: Divergent Brain Network Activity in Asymptomatic C9orf72 and SOD1 Variant Carriers Compared With Established Amyotrophic Lateral Sclerosis
Source: Hum Brain Mapp. 2025 Oct 3;46(14):e70345. doi: 10.1002/hbm.70345 (PMC12492477; doi:10.1002/hbm.70345)
Supplement: Supplementary file 1 — Figure S1: General linear model design—group comparison. Design matrix used to predict network metrics. The first regressor (HC) models the mean value of the network metric across healthy controls. The second regressor models the mean value of the network metric across symALS patients. The third and fourth (aC9 and aSOD) model the mean values for each asymptomatic carrier group respectively. The remaining regressors are included to model known sources of variability (age, sex, missing structural) across participants. This has the effect of minimising the impact of these confounds on the group means. The confound regressors are calculated by z‐transforming the values for age, sex (1 = female or 2 = male) and missing structural (1 = not missing, 2 = missing) across participants. Figure S2: Explanation of spectral shape metrics. The top graph shows an example annotated power spectrum density curve (PSD). The red line represents the FOOOF full model fit. The blue dashed line represents the aperiodic component. The 1/f exponent is derived by taking the steepness of the slope of the aperiodic fit. The bottom graph shows the same PSD after the aperiodic fit has been subtracted from the full model fit, and therefore represents an estimation of the periodic component. The CoE is derived by calculating the frequency at which the sum of the power below = the sum of the power above (represented by the red line). Spectral slowing would be represented by a shift of the CoE to the left. Figure S3: (A) The power profile. Oscillatory power in each disease group compared to healthy controls. symALS was characterised by reduced beta and increased high‐gamma power in motor regions. aC9 similarly showed decreased beta power in occipital and temporal regions, but also showed increased frontal theta, and increased alpha in motor and occipital regions. aSOD showed an increase in frontal gamma, but a decrease in occipital gamma and high‐gamma, and a decrease in frontal theta. (B) Beta power [file HBM-46-e70345-s002.pdf]

Supplementary material

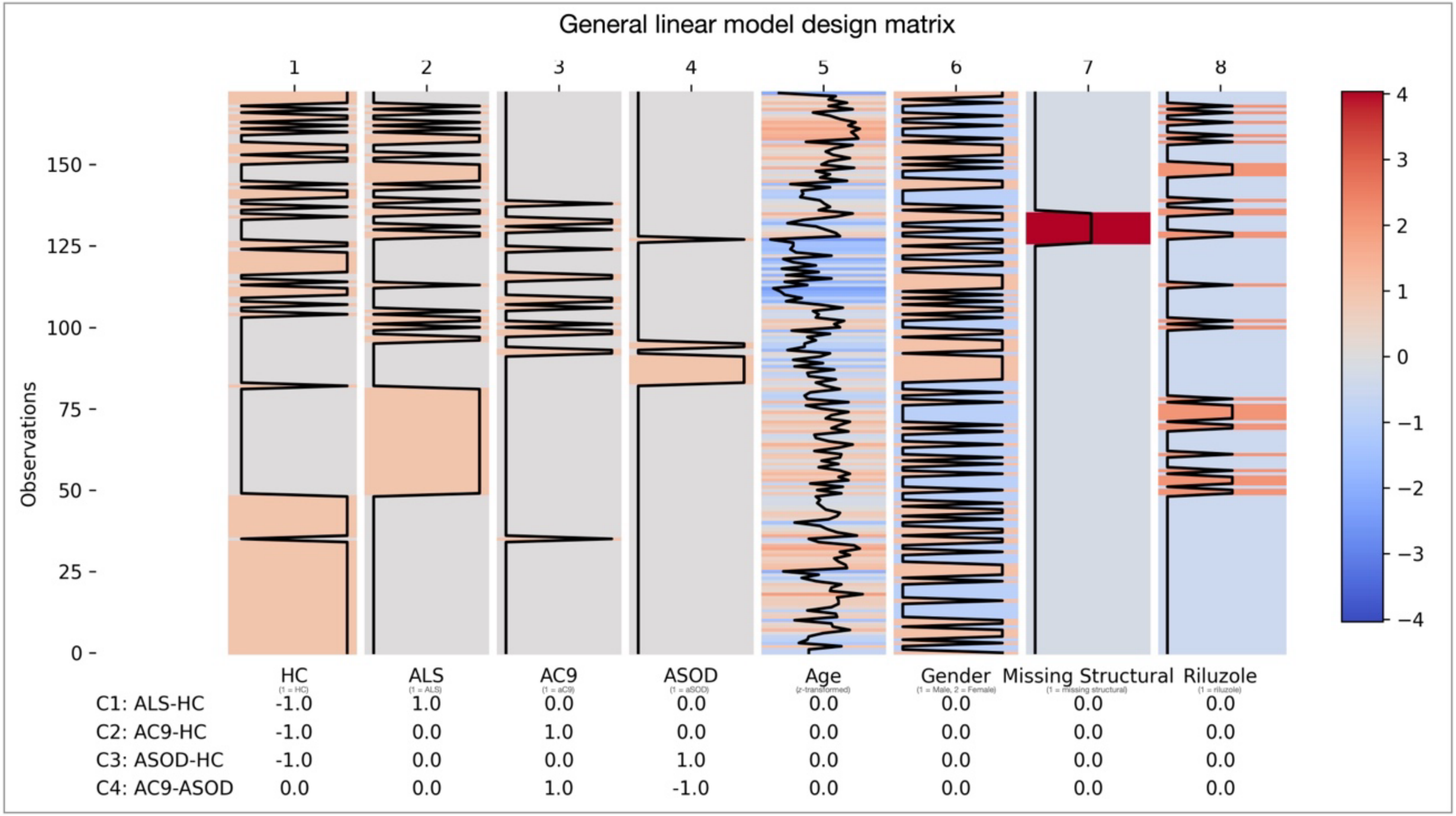

Supplementary Figure 1 - General linear model design – group comparison. Design matrix used to predict network metrics. The first regressor (HC) models the mean value of the network metric across healthy controls. The second regressor models the mean value of the

network metric across symALS patients. The third and fourth (aC9 and aSOD) model the mean values for each asymptomatic carrier group respectively. The remaining regressors are included to model known sources of variability (age, sex, missing structural) across participants. This has the effect of minimising the impact of these confounds on the group means. The confound regressors are calculated by z-transforming the values for age, sex (1=female or 2=male) and missing structural (1 = not missing, 2 = missing) across participants.

The power profile (results continued)

High-gamma power was increased in the motor areas of symALS (right premotor: ($t(159) = 2.648$, $p = 0.014$)) and reduced in occipital regions of aSOD (right visual: ($t(159) = -2.431$, $p = 0.026$)) (**Supplementary Figure 2 – bottom right)**. Frontal gamma was increased (right inferior frontal: ($t(159) = 2.259$, $p = 0.044$)) and theta was decreased (right inferior frontal: ($t(159) = -2.865$, $p = 0.003$)) in aSOD (**Supplementary Figure 2 – middle right)**. Both aC9 and aSOD groups showed occipital increases in alpha power (right visual $t(159) = 2.371$ and 2.359 respectively, $p = 0.03$) (**Supplementary Figure 2 – bottom left)**. Frontal theta power was increased in aC9 compared to the aSOD group (inferior frontal left: ($t(159) = 3.181$, $p = 0.042$)) (**Supplementary Figure 2 – middle right)**.

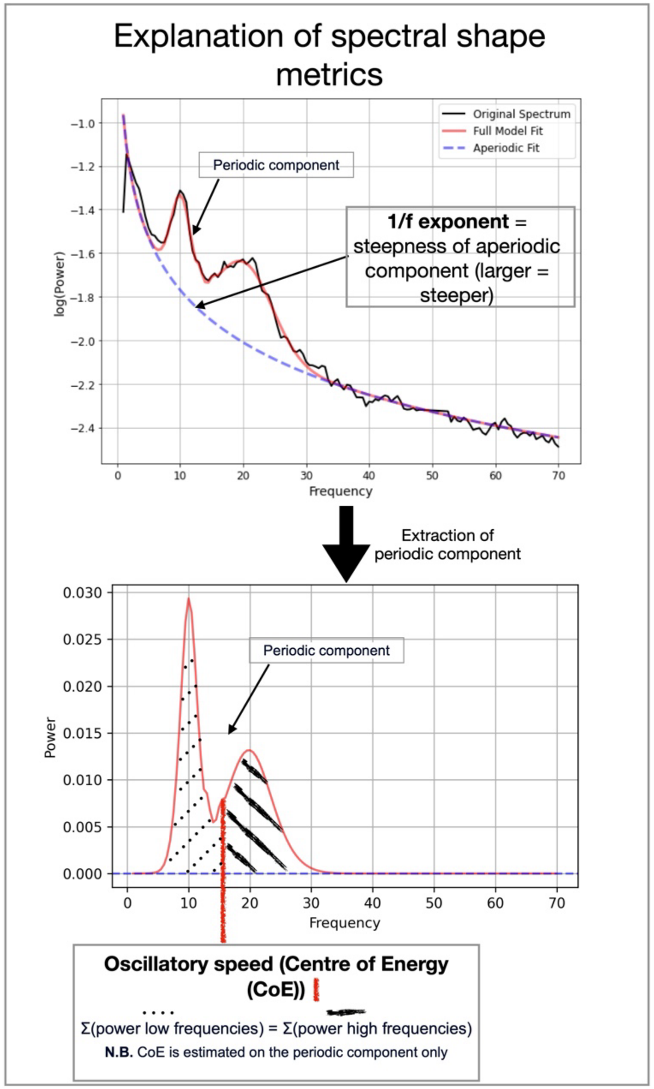

Supplementary Figure 2 - Explanation of spectral shape metrics. The top graph shows an example annotated power spectrum density curve (PSD). The red line represents the FOOOF full model fit. The blue dashed line represents the aperiodic component. The 1/f exponent is derived by taking the steepness of the slope of the aperiodic fit. The bottom graph shows the same PSD after the aperiodic fit has been subtracted from the full model fit, and therefore represents an estimation of the periodic component. The CoE is derived by calculating the frequency at which the sum of the power below = the sum of the power above (represented by the red line). Spectral slowing would be represented by a shift of the CoE to the left.

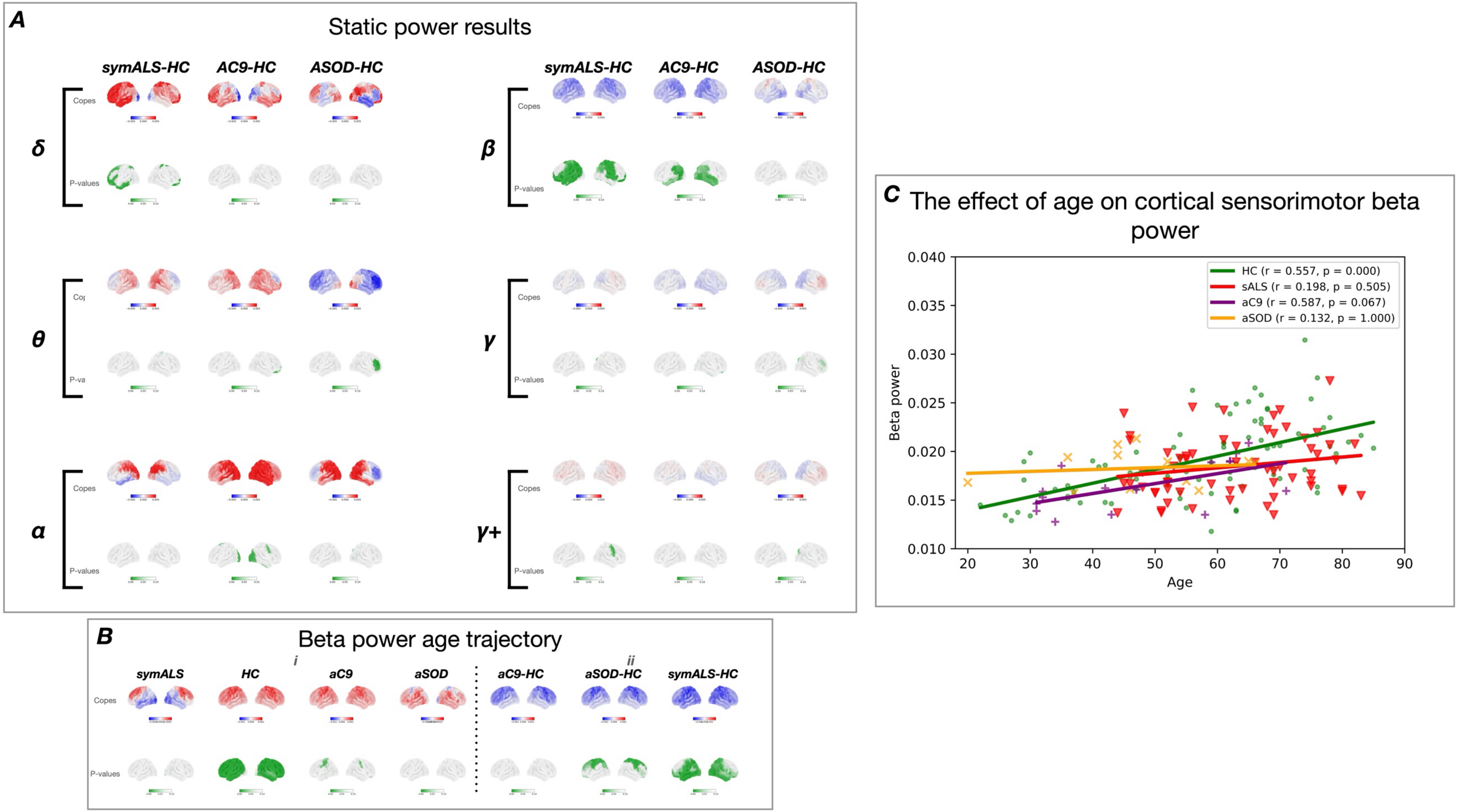

Supplementary Figure 3 – *A* - The power profile. Oscillatory power in each disease group compared to healthy controls. symALS was characterised by reduced beta and increased high-gamma power in motor regions. aC9 similarly showed decreased beta power in occipital and

temporal regions, but also showed increased frontal theta, and increased alpha in motor and occipital regions. aSOD showed an increase in frontal gamma, but a decrease in occipital gamma and high-gamma, and a decrease in frontal theta. ***B* – Beta power age trajectory.** ***i*** – shows the effect of age on cortical beta power. Most brain regions in HC showed a significant increase in beta power with age. Sensorimotor regions showed a significant increase of beta power with age in aC9. ***ii*** – Shows the parcel-wise difference in age trajectories between disease groups. Both symALS and aSOD showed a significantly reduced effect of age in sensorimotor regions compared to HC. ***C* - Beta power and age.** Linear regression to show the effect of age in each disease group. Mean beta power from the 29 parcels which had a $p<0.001$ effect of age in the HC cohort were extracted from each participant and plotted here. Each data point represents the mean beta power across 29 parcels in each participant. The regression lines were calculated by performing linear regression on the mean beta power across the 29 regions. P-values are displayed after Bonferroni correction for multiple comparisons. Beta power generally increased with age in the HC cohort ($r = 0.557$, $p < 0.001$). Deviation from healthy (a reduction in beta power) occurred between 50 and 60 years of age in symALS. aSOD showed higher, whilst aC9 showed lower beta power when compared to controls prior to reaching their 50s. By the time they reached their 60s, both genetically at-risk groups aligned with the symALS group, having come from different ‘sides’ of the healthy beta range.

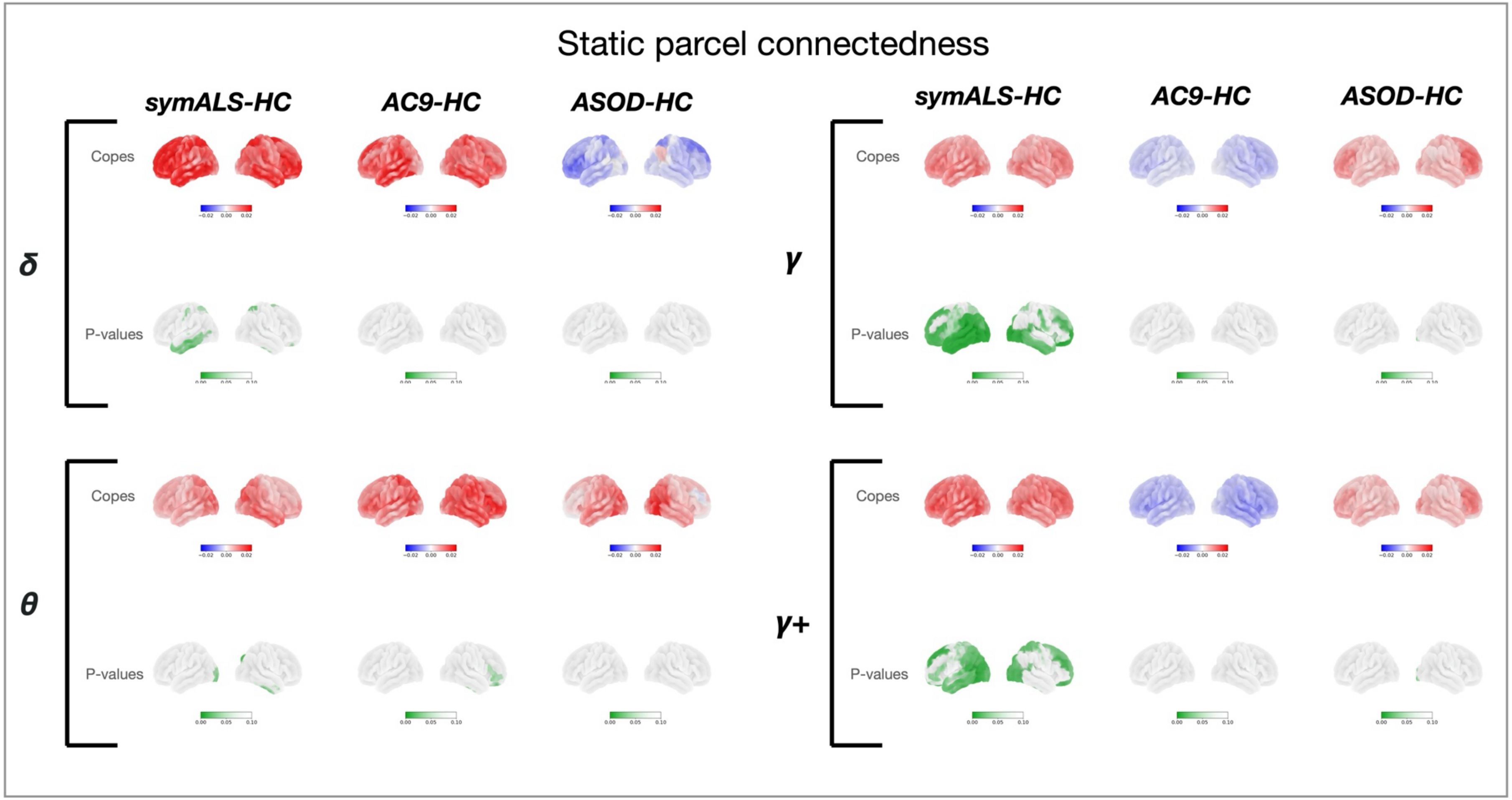

Supplementary Figure 4 – Static parcel connectedness profile. Per parcel functional connectivity between each parcel and the rest of the brain was increased in fronto-temporal regions in delta, gamma and high-gamma frequency bands in symALS compared to controls. aC9 and aSOD showed no significant changes in the delta band compared to controls. aC9 showed an increase in static parcel connectedness in right

fronto-temporal regions in the theta band. aSOD showed a small area of increase in the right occipital parcel compared to controls in the high-gamma band.

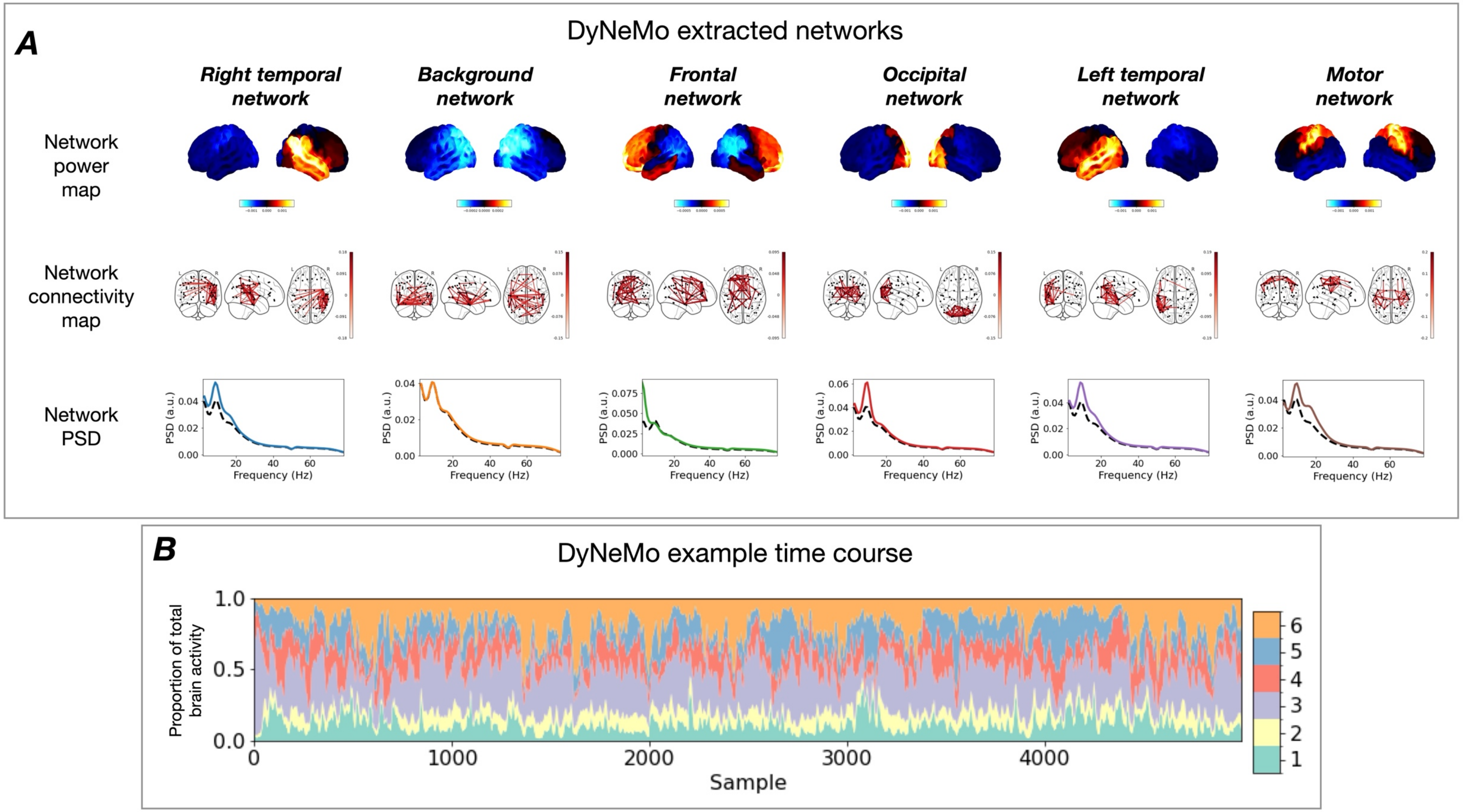

Supplementary Figure 5 – *A* - DyNeMo network descriptions. The first two rows show the power maps across participants of the six networks extracted from DyNeMo and the associated connectivity maps (strongest 5% of connections) respectively. The third row shows the

associated network PSDs with the black dotted line representing the mean PSD across networks and the coloured line representing the network-specific PSD. ***B* - DyNeMo example time course.** Shows an example of one participant's DyNeMo time course. The proportion of total brain activity explained by each mode (1-6) varies over time. This proportion is represented by the thickness of each colour at each time point.

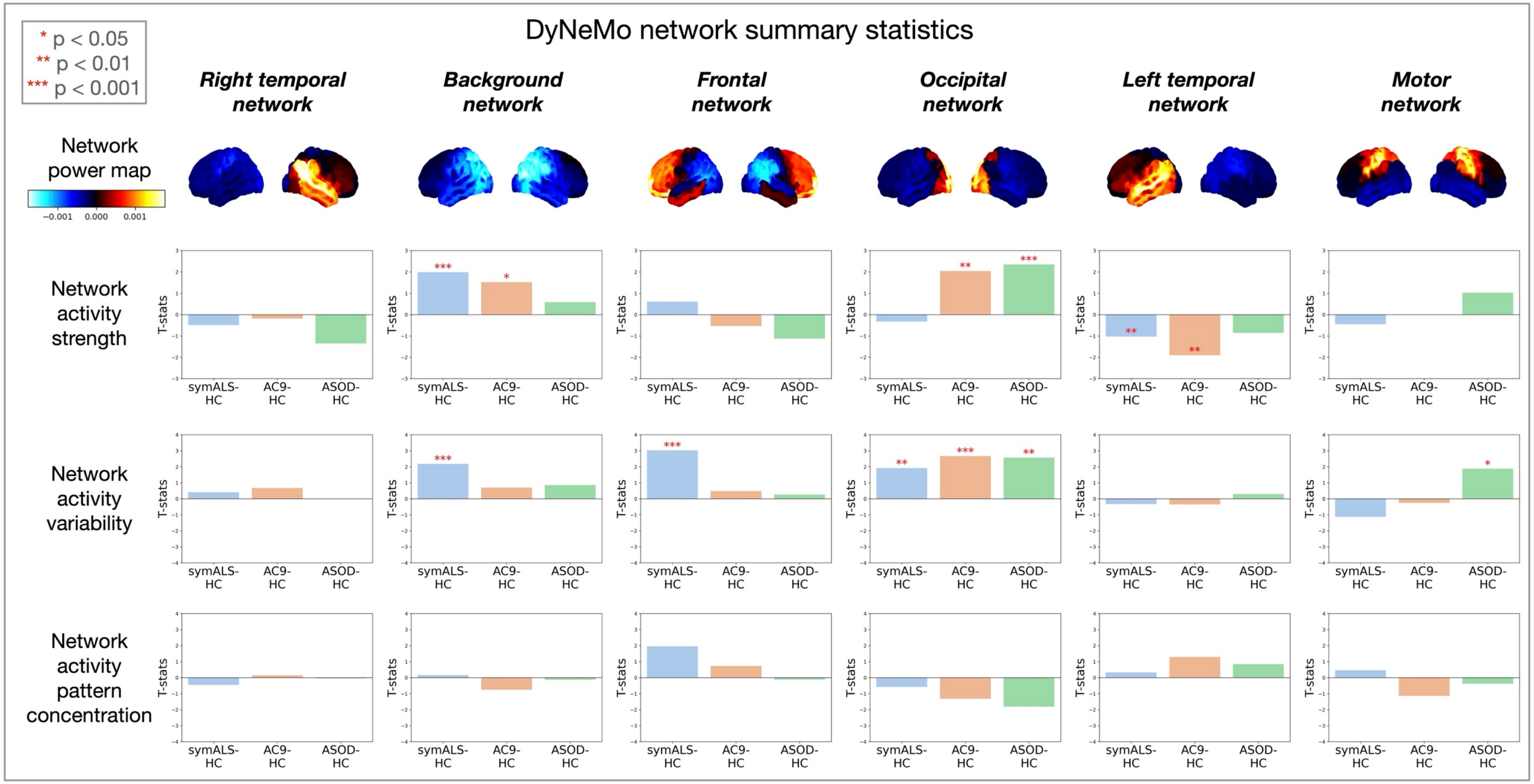

Supplementary Figure 6 – DyNeMo network summary statistics. The first row shows the power maps across participants of networks extracted from DyNeMo. The last three rows show group-comparisons from a General Linear Model comparing network activity strength, network activity variability (standard deviation) and network activity pattern concentration (kurtosis) respectively for each network. Maximum t-statistic correction for multiple comparisons was applied across networks. Compared to controls (HC), people with amyotrophic lateral sclerosis

(symALS) showed decreased network activity strength in the left temporal network but increased network activity strength in the background network. The network activity variability was increased in symALS in the frontal, occipital and background networks. Asymptomatic *C9orf72* carriers (aC9) showed an increase in occipital and background network activity strength and a decrease in left temporal network activity strength. aC9 occipital network activity variability was increased. Asymptomatic *SOD1* carriers (aSOD) showed increased occipital network activity strength and decreased right temporal network activity strength. The occipital and motor networks both had a higher activity variability in aSOD.

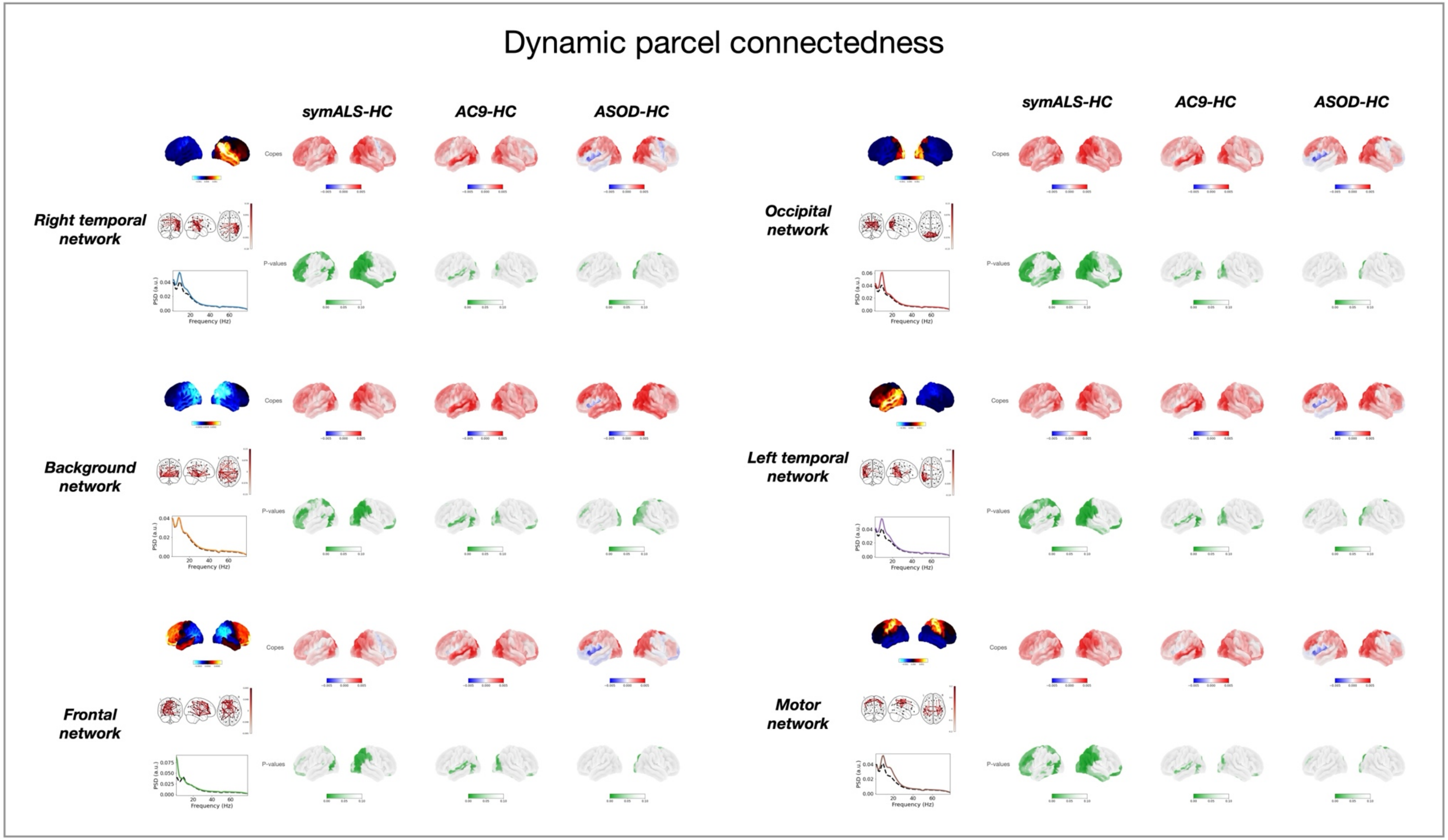

Supplementary Figure 7 – DyNeMo network connectivity comparisons. Shows GLM results from group-level comparisons of global connectivity in each network. symALS showed increased dynamic (intra-network) parcel connectedness in all networks in all regions other than the motor cortex. aC9 and aSOD showed smaller areas of significant increase in all networks in frontal, temporal and occipital regions.

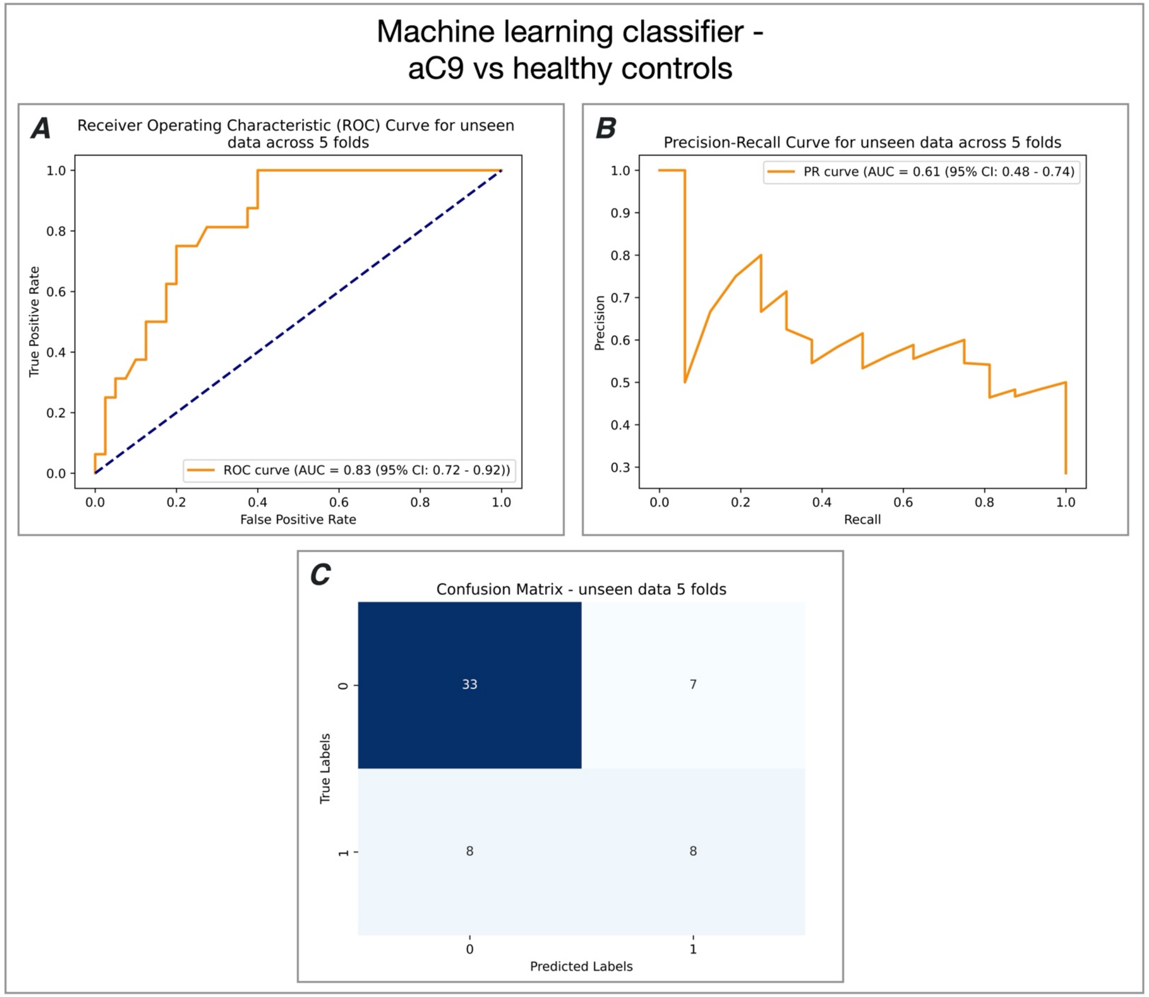

Supplementary Figure 8 – Random Forest Classifier performance predicting aC9 from matched HC. *A* – ROC curve for unseen predictions across 5 folds. The Random Forest Classifier achieved a ROC AUC of 0.83. ***B* – Confusion matrix.** Shows classifier performance. C – Precision-recall (PR) curve. Shows a classifier performance PR AUC of 0.61.

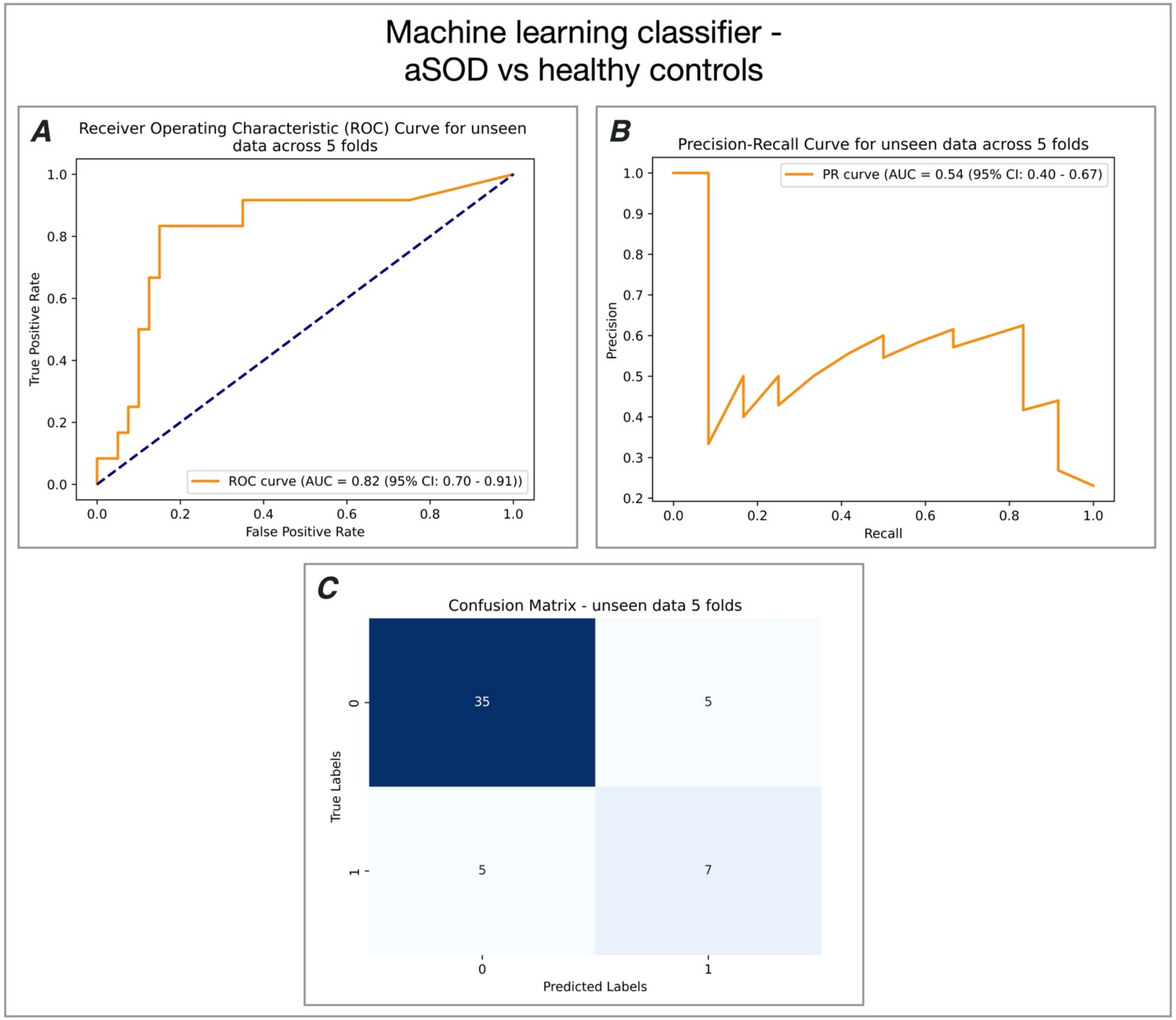

Supplementary Figure 9 – Random Forest Classifier performance predicting aSOD from matched HC. *A* – ROC curve for unseen predictions across 5 folds. The Random Forest Classifier achieved a ROC AUC of 0.82. ***B* – Confusion matrix.** Shows classifier performance.

C – Precision-recall (PR) curve. Shows a classifier performance PR AUC of 0.54.

Supplementary Table 1: DyNeMo hyperparameter settings

DyNeMo Function	Hyperparameters	Setting
Pepare data	n embeddings	15
	n pca components	100
Train DyNeMo	n modes	6,7,8
	Learn means	False
	Learn covariances	True
	n kl annealing epochs	10
	n epochs	20
	Learning rate	0.001
Initialise	n init	10
	n epochs	2

Supplementary Table 2: DyNeMo Free Energy Scores. 30 models were trained for each number of modes. The run with the lowest free energy from each mode selection is displayed here.

**N modes**	**Lowest free energy**
6	27506.283203125
7	27474.115234375
8	27448.431640625

Supplementary Table 3: Glasser52 parcellation co-ordinates. Parcel names and MNE-coordinates of parcel centres of the Glasser52 Parcellation.

Region Index	Location description	X	Y	Z
0	Primary and Early Visual Cortex Right	14.5	-80.5	-0.4
1	Dorsal Stream Visual Cortex Right	19.4	-81.4	30.6
2	Ventral Stream Visual Cortex Right	29.9	-57.6	-17.7
3	MT+ Complex and Neighbouring Visual Areas Right	42.2	-71.5	0.0
4	Superior Somatosensory and Motor Cortex Right	23.6	-29.5	61.0
5	Inferior Somatosensory and Motor Cortex Right	49.1	-13.4	38.3
6	Supplementary Motor Area Right	12.7	-2.2	62.9
7	Cingulate Motor Areas & Area 5 Right	10.0	-31.2	53.6
8	Premotor Cortex Right	40.9	0.9	41.1
9	Insular & Frontoparietal Operculum Right	37.7	1.6	4.0
10	Early Auditory Cortex Right	40.2	-27.5	13.5
11	Auditory Association Cortex Right	55.0	-14.0	-6.5
12	Medial Temporal Cortex Right	25.0	-22.1	-22.1
13	Lateral Temporal Cortex Right	48.5	-16.3	-25.3
14	Temporal-Parieto-Occipital Junction Right	52.1	-48.1	12.3
15	Medial Bank of the Intra-parietal Sulcus Right	29.5	-51.0	43.0
16	Superior Medial Parietal Cortex Right	19.1	-58.4	59.6
17	Inferior Parietal Cortex Task-Positive Network Right	56.7	-29.7	35.7
18	Inferior Parietal Cortex Task-Negative Network Right	47.4	-56.1	35.8
19	Intraparietal Sulcus & PGP Right	36.5	-71.1	32.6
20	Posterior Cingulate Cortex Right	10.3	-55.1	26.8
21	Anterior Cingulate and Medial Prefrontal Cortex Right	6.1	33.8	14.2
22	Orbital and Polar Frontal Cortex Right	17.1	47.0	-11.6
23	Inferior Frontal Cortex Right	45.4	32.5	3.8
24	Inferior Dorsolateral Prefrontal Cortex Right	34.8	37.1	25.7
25	Superior Dorsolateral Prefrontal Cortex Right	21.0	31.9	45.9
26	Primary and Early Visual Cortex Left	-16.9	-82.0	-0.9
27	Dorsal Stream Visual Cortex Left	-21.7	-84.3	27.0
28	Ventral Stream Visual Cortex Left	-34.5	-56.0	-18.6
29	MT+ Complex and Neighbouring Visual Areas Left	-45.2	-70.3	-1.4
30	Superior Somatosensory and Motor Cortex Left	-22.6	-30.4	62.8

31	Inferior Somatosensory and Motor Cortex Left	-48.3	-18.7	41.7
32	Supplementary Motor Area Left	-14.0	-2.7	62.7
33	Cingulate Motor Areas & Area 5 Left	-14.4	-30.6	49.7
34	Premotor Cortex Left	-40.3	-1.7	43.8
35	Insular & Frontoparietal Operculum Left	-41.3	0.1	3.7
36	Early Auditory Cortex Left	-45.4	-29.4	12.1
37	Auditory Association Cortex Left	-55.3	-17.3	-7.8
38	Medial Temporal Cortex Left	-27.9	-22.2	-22.6
39	Lateral Temporal Cortex Left	-49.6	-17.9	-25.1
40	Temporal-Parieto-Occipital Junction Left	-53.1	-54.0	14.5
41	Medial Bank of the Intra-parietal Sulcus Left	-31.1	-50.0	41.1
42	Superior Medial Parietal Cortex Left	-21.3	-60.5	58.2
43	Inferior Parietal Cortex Task-Positive Network Left	-56.6	-36.3	36.6
44	Inferior Parietal Cortex Task-Negative Network Left	-45.1	-63.4	35.2
45	Intraparietal Sulcus & PGP Left	-36.7	-70.0	30.4
46	Posterior Cingulate Cortex Left	-11.6	-49.5	28.0
47	Anterior Cingulate and Medial Prefrontal Cortex Left	-8.7	32.0	11.4
48	Orbital and Polar Frontal Cortex Left	-21.9	46.3	-8.1
49	Inferior Frontal Cortex Left	-45.1	28.2	7.7
50	Inferior Dorsolateral Prefrontal Cortex Left	-37.2	36.2	25.2
51	Superior Dorsolateral Prefrontal Cortex Left	-23.5	28.6	45.7
