# Supplementary material for: Divergent Brain Network Activity in Asymptomatic C9orf72 and SOD1 Variant Carriers Compared With Established Amyotrophic Lateral Sclerosis
Source: Hum Brain Mapp. 2025 Oct 3;46(14):e70345. doi: 10.1002/hbm.70345 (PMC12492477; doi:10.1002/hbm.70345)
Supplement: Supplementary file 2 — Table S4: Full table of static results. Summarizes statistical analysis and results for each measure of cortical activity in the static analysis. Table S5: Full table of dynamic results. Summarizes statistical analysis and results for each measure of cortical activity in the dynamic analysis. [file HBM-46-e70345-s001.pdf]

Supplementary Table 4: Full table of static results. Summarises statistical analysis and results for each measure of cortical activity in the static analysis.

1

Metric	Comparison (0 = symsymALS-HC, 1 = PC9-HC, 2 = PSOD-HC, 3 = PC9-symALS)	Frequency (0 = delta, 1 = theta, 2 = alpha, 3 = beta, 4 = gamma, 5 = high-gamma)	Region (for labels please see Glasser52 labels table)	Degrees of freedom	T statistic	P value
POWER	0	0	4	157	2.754	0.006
	0	0	6	157	2.587	0.015
	0	0	22	157	2.945	0.001
	0	0	39	157	3.058	0.001
	0	0	42	157	2.532	0.018
	0	0	48	157	2.898	0.002
	0	0	49	157	2.913	0.002
	0	0	51	157	2.532	0.018
	0	3	2	157	-2.399	0.034
	0	3	4	157	-2.985	0.001
	0	3	5	157	-2.963	0.001
	0	3	6	157	-2.61	0.013
	0	3	7	157	-3.496	0
	0	3	8	157	-2.86	0.003
	0	3	9	157	-3.228	0
	0	3	10	157	-2.674	0.009
	0	3	11	157	-2.815	0.004
	0	3	16	157	-2.602	0.014
	0	3	17	157	-2.797	0.005
	0	3	18	157	-2.407	0.033
	0	3	20	157	-2.716	0.008
	0	3	21	157	-2.371	0.039
	0	3	22	157	-3.128	0.001
	0	3	27	157	-2.749	0.006
	0	3	29	157	-3.112	0.001
	0	3	31	157	-2.991	0.001
	0	3	32	157	-2.981	0.001
	0	3	33	157	-3.422	0
	0	3	35	157	-3.022	0.001
	0	3	36	157	-2.846	0.003
	0	3	37	157	-2.73	0.007
	0	3	39	157	-3.082	0.001
	0	3	40	157	-2.474	0.024
	0	3	41	157	-3.208	0.001

2

	0	3	42	157	-3.914	0
	0	3	43	157	-3.233	0
	0	3	44	157	-2.999	0.001
	0	3	45	157	-3.153	0.001
	0	3	46	157	-3.455	0
	0	3	47	157	-2.706	0.008
	0	3	48	157	-3.556	0
	0	3	49	157	-2.615	0.013
	0	4	1	157	-2.351	0.043
	0	5	7	157	2.37	0.039
	0	5	8	157	2.78	0.005
	0	5	9	157	2.334	0.047
	1	2	0	157	2.642	0.013
	1	2	1	157	2.376	0.044
	1	2	2	157	2.392	0.04
	1	2	3	157	2.587	0.017
	1	2	8	157	2.585	0.017
	1	2	19	157	2.871	0.005
	1	2	20	157	2.425	0.036
	1	2	26	157	2.532	0.022
	1	2	27	157	2.969	0.003
	1	2	32	157	2.648	0.013
	1	3	1	157	-2.529	0.023
	1	3	2	157	-3.432	0
	1	3	3	157	-2.715	0.011
	1	3	11	157	-2.694	0.011
	1	3	13	157	-2.458	0.032
	1	3	14	157	-2.422	0.036
	1	3	15	157	-2.568	0.019
	1	3	17	157	-2.505	0.025
	1	3	18	157	-2.381	0.043
	1	3	20	157	-2.444	0.033
	1	3	27	157	-2.975	0.003
	1	3	28	157	-2.778	0.009
	1	3	29	157	-3.244	0.001
	1	3	38	157	-2.561	0.019
	1	3	40	157	-2.763	0.009
	1	3	41	157	-2.969	0.003
	1	3	43	157	-3.023	0.002
	1	3	44	157	-2.515	0.024
	1	3	45	157	-3.027	0.002
	1	3	46	157	-2.558	0.019

3

	2	1	23	157	-2.809	0.007
	2	1	24	157	-3.331	0.001
	2	2	1	157	2.391	0.048
	2	4	1	157	-2.417	0.043
	2	5	1	157	-2.477	0.033
	3	1	22	157	3.169	0.044
	3	1	23	157	3.149	0.046
beta_power_age	HC	3	2	157	3.159	0.028
	HC	3	3	157	3.378	0.011
	HC	3	4	157	5.266	0.001
	HC	3	5	157	5.62	0.001
	HC	3	6	157	5.544	0.001
	HC	3	7	157	6.764	0
	HC	3	8	157	5.97	0.001
	HC	3	9	157	4.969	0.001
	HC	3	10	157	4.778	0.001
	HC	3	11	157	2.911	0.045
	HC	3	12	157	4.137	0.001
	HC	3	13	157	3.752	0.003
	HC	3	14	157	3.425	0.01
	HC	3	15	157	4.367	0.001
	HC	3	16	157	5.085	0.001
	HC	3	17	157	4.166	0.001
	HC	3	18	157	4.342	0.001
	HC	3	19	157	3.686	0.003
	HC	3	20	157	4.683	0.001
	HC	3	21	157	5.151	0.001
	HC	3	22	157	4.175	0.001
	HC	3	23	157	4.517	0.001
	HC	3	24	157	4.647	0.001
	HC	3	25	157	5.326	0.001
	HC	3	29	157	3.591	0.005
	HC	3	30	157	5.029	0.001
	HC	3	31	157	5.826	0.001
	HC	3	32	157	5.521	0.001
	HC	3	33	157	6.352	0.001
	HC	3	34	157	5.821	0.001
	HC	3	35	157	4.821	0.001
	HC	3	36	157	5.516	0.001
	HC	3	37	157	3.618	0.005
	HC	3	38	157	4.069	0.001

4

	HC	3	39	157	3.577	0.005
	HC	3	40	157	3.546	0.006
	HC	3	41	157	5.477	0.001
	HC	3	42	157	5.685	0.001
	HC	3	43	157	4.82	0.001
	HC	3	44	157	4.759	0.001
	HC	3	45	157	4.421	0.001
	HC	3	46	157	4.716	0.001
	HC	3	47	157	4.839	0.001
	HC	3	48	157	4.383	0.001
	HC	3	49	157	4.71	0.001
	HC	3	50	157	4.862	0.001
	HC	3	51	157	5.16	0.001
	aC9	3	5	157	3.293	0.029
	aC9	3	31	157	3.377	0.023
	aC9	3	33	157	3.219	0.037
	aSOD-HC	3	4	157	-4.065	0.005
	aSOD-HC	3	5	157	-3.667	0.011
	aSOD-HC	3	6	157	-3.687	0.01
	aSOD-HC	3	7	157	-4.467	0.004
	aSOD-HC	3	8	157	-4.346	0.004
	aSOD-HC	3	16	157	-3.661	0.013
	aSOD-HC	3	21	157	-3.166	0.039
	aSOD-HC	3	25	157	-3.683	0.011
	aSOD-HC	3	30	157	-3.517	0.02
	aSOD-HC	3	31	157	-3.74	0.008
	aSOD-HC	3	32	157	-4.082	0.005
	aSOD-HC	3	33	157	-4.096	0.005
	aSOD-HC	3	34	157	-4.556	0.003
	aSOD-HC	3	42	157	-3.661	0.013
	aSOD-HC	3	44	157	-3.104	0.044
	aSOD-HC	3	46	157	-3.489	0.022
	aSOD-HC	3	51	157	-3.214	0.037
	symALS-HC	3	0	157	-3.453	0.014
	symALS-HC	3	1	157	-3.217	0.027
	symALS-HC	3	2	157	-3.963	0.002
	symALS-HC	3	3	157	-3.761	0.005
	symALS-HC	3	4	157	-3.29	0.023
	symALS-HC	3	7	157	-4.328	0
	symALS-HC	3	9	157	-3.25	0.024
	symALS-HC	3	10	157	-3.614	0.009
	symALS-HC	3	13	157	-3.522	0.013

	symALS-HC	3	14	157	-3.346	0.019
	symALS-HC	3	15	157	-3.101	0.036
	symALS-HC	3	16	157	-3.596	0.01
	symALS-HC	3	17	157	-3.483	0.013
	symALS-HC	3	18	157	-3.152	0.03
	symALS-HC	3	19	157	-3.681	0.008
	symALS-HC	3	20	157	-3.971	0.002
	symALS-HC	3	26	157	-3.188	0.027
	symALS-HC	3	28	157	-3.303	0.021
	symALS-HC	3	29	157	-3.802	0.004
	symALS-HC	3	31	157	-3.635	0.009
	symALS-HC	3	33	157	-4.016	0.002
	symALS-HC	3	35	157	-3.822	0.004
	symALS-HC	3	36	157	-4.213	0.001
	symALS-HC	3	37	157	-4.189	0.001
	symALS-HC	3	38	157	-3.84	0.003
	symALS-HC	3	39	157	-3.844	0.003
	symALS-HC	3	41	157	-3.964	0.002
	symALS-HC	3	42	157	-4.112	0.001
	symALS-HC	3	43	157	-3.98	0.002
	symALS-HC	3	44	157	-3.362	0.018
	symALS-HC	3	45	157	-3.549	0.011
	symALS-HC	3	46	157	-3.896	0.003
	symALS-HC	3	47	157	-3.095	0.037
	symALS-HC	3	48	157	-3.366	0.018
**COE**	0		0	157	-2.065	0.003
	0		1	157	-2.791	0.001
	0		2	157	-2.369	0.001
	0		3	157	-2.014	0.004
	0		4	157	-2.225	0.001
	0		5	157	-2.032	0.004
	0		6	157	-2.166	0.002
	0		7	157	-2.266	0.001
	0		8	157	-1.904	0.006
	0		9	157	-2.309	0.001
	0		10	157	-2.09	0.003
	0		11	157	-3.107	0
	0		12	157	-1.98	0.005
	0		14	157	-2.536	0.001
	0		15	157	-2.239	0.001
	0		16	157	-2.119	0.002
	0		17	157	-2.322	0.001

	0		18	157	-2.579	0.001
	0		19	157	-2.64	0.001
	0		20	157	-2.471	0.001
	0		21	157	-2.121	0.002
	0		23	157	-1.825	0.01
	0		25	157	-3.038	0.001
	0		26	157	-1.592	0.04
	0		27	157	-3.073	0.001
	0		30	157	-2.724	0.001
	0		31	157	-2.369	0.001
	0		32	157	-2.112	0.002
	0		33	157	-2.421	0.001
	0		35	157	-2.214	0.001
	0		36	157	-2.631	0.001
	0		37	157	-2.51	0.001
	0		38	157	-1.908	0.006
	0		39	157	-1.98	0.005
	0		40	157	-2.256	0.001
	0		41	157	-3.055	0.001
	0		42	157	-2.76	0.001
	0		43	157	-2.892	0.001
	0		44	157	-2.501	0.001
	0		45	157	-3.035	0.001
	0		46	157	-3.026	0.001
	0		49	157	-1.596	0.038
	1		0	157	-2.684	0.003
	1		1	157	-3.637	0.001
	1		2	157	-3.71	0.001
	1		3	157	-2.81	0.002
	1		4	157	-4.177	0.001
	1		5	157	-3.345	0.001
	1		7	157	-2.947	0.001
	1		8	157	-3.294	0.001
	1		9	157	-2.741	0.002
	1		10	157	-2.414	0.004
	1		11	157	-2.747	0.002
	1		13	157	-2.392	0.004
	1		14	157	-3.408	0.001
	1		15	157	-4.188	0
	1		16	157	-4.086	0.001
	1		17	157	-3.323	0.001
	1		18	157	-3.548	0.001

	1		19	157	-3.727	0.001
	1		20	157	-3.516	0.001
	1		25	157	-2.423	0.004
	1		26	157	-2.663	0.003
	1		27	157	-4.096	0.001
	1		28	157	-2.35	0.004
	1		29	157	-2.785	0.002
	1		30	157	-3.169	0.001
	1		31	157	-3.677	0.001
	1		32	157	-2.906	0.001
	1		33	157	-3.393	0.001
	1		34	157	-2.856	0.002
	1		35	157	-2.002	0.024
	1		36	157	-2.435	0.003
	1		37	157	-2.127	0.018
	1		38	157	-2.859	0.002
	1		39	157	-2.24	0.007
	1		40	157	-2.952	0.001
	1		41	157	-3.656	0.001
	1		42	157	-1.886	0.038
	1		43	157	-2.631	0.003
	1		44	157	-3.332	0.001
	1		45	157	-3.973	0.001
	1		46	157	-3.533	0.001
	1		47	157	-2.045	0.023
	1		49	157	-1.972	0.027
	1		50	157	-1.988	0.026
	2		6	157	1.918	0.032
	2		23	157	2.304	0.01
	2		24	157	2.527	0.004
	2		26	157	-1.997	0.022
	2		27	157	-2.031	0.021
	2		34	157	1.851	0.041
	2		39	157	-2.124	0.017
	2		50	157	2.399	0.008
	3		4	157	-3.909	0
	3		5	157	-3.014	0.012
	3		6	157	-2.898	0.022
	3		7	157	-2.816	0.027
	3		8	157	-3.551	0.003
	3		16	157	-2.652	0.037
	3		23	157	-3.046	0.011

	3		24	157	-3.153	0.006
	3		25	157	-3.116	0.006
	3		30	157	-3.155	0.006
	3		32	157	-3.489	0.003
	3		33	157	-2.851	0.024
	3		34	157	-3.64	0.001
	3		49	157	-2.883	0.023
	3		50	157	-3.458	0.003
**1F**	0		8	157	-1.896	0.014
	0		9	157	-1.699	0.035
	1		12	157	2.054	0.024
	1		13	157	1.944	0.034
	1		22	157	1.98	0.03
	1		28	157	1.84	0.046
	1		38	157	2.076	0.023
	2		0	157	2.526	0.002
	2		1	157	2.628	0.002
	2		3	157	1.962	0.026
	2		15	157	2.033	0.018
	2		18	157	2.611	0.002
	2		19	157	2.716	0
	2		20	157	1.782	0.049
	2		24	157	-2.151	0.009
	2		26	157	1.818	0.046
	2		27	157	2.298	0.004
	2		45	157	1.919	0.032
**AEC**	0	0	16	157	2.741	0.035
	0	0	39	157	2.643	0.049
	0	1	1	157	2.979	0.016
	0	4	0	157	3.25	0.005
	0	4	1	157	2.832	0.026
	0	4	2	157	3.181	0.007
	0	4	3	157	3.066	0.012
	0	4	5	157	2.833	0.026
	0	4	10	157	2.764	0.032
	0	4	12	157	2.736	0.035
	0	4	13	157	2.715	0.04
	0	4	14	157	2.82	0.027
	0	4	20	157	3.145	0.009
	0	4	21	157	2.708	0.041
	0	4	22	157	3.176	0.008
	0	4	24	157	2.851	0.025

	0	4	26	157	3.22	0.006
	0	4	27	157	2.968	0.016
	0	4	28	157	3.55	0.001
	0	4	29	157	3.266	0.005
	0	4	36	157	3.393	0.003
	0	4	37	157	3.301	0.004
	0	4	38	157	2.786	0.03
	0	4	39	157	3.323	0.004
	0	4	40	157	3.439	0.003
	0	4	43	157	3.408	0.003
	0	4	44	157	3.074	0.012
	0	4	45	157	2.688	0.044
	0	4	47	157	2.841	0.025
	0	4	48	157	2.779	0.031
	0	4	49	157	2.976	0.016
	0	4	51	157	2.899	0.021
	0	5	0	157	2.779	0.031
	0	5	1	157	2.884	0.022
	0	5	2	157	2.915	0.019
	0	5	3	157	2.817	0.028
	0	5	4	157	2.652	0.048
	0	5	6	157	2.66	0.047
	0	5	7	157	2.826	0.027
	0	5	10	157	2.649	0.049
	0	5	14	157	2.703	0.042
	0	5	16	157	2.793	0.029
	0	5	19	157	2.721	0.038
	0	5	20	157	2.802	0.029
	0	5	22	157	2.67	0.046
	0	5	24	157	2.904	0.02
	0	5	25	157	2.679	0.045
	0	5	26	157	2.711	0.041
	0	5	27	157	3.025	0.014
	0	5	28	157	3.21	0.006
	0	5	29	157	3.09	0.012
	0	5	36	157	2.653	0.048
	0	5	39	157	2.728	0.037
	0	5	40	157	3.065	0.012
	0	5	41	157	2.733	0.036
	0	5	42	157	2.712	0.04
	0	5	43	157	2.717	0.04
	0	5	44	157	2.757	0.033

	0	5	45	157	3.032	0.014
	0	5	47	157	2.953	0.017
	0	5	51	157	3.102	0.011

Supplementary Table 5: Full table of dynamic results. Summarises statistical analysis and results for each measure of cortical activity in the dynamic analysis.

Metric<br><br>1	Comparison (0 = ALS-HC, 1 = PC9-HC, 2 = PSOD-HC, 3 = PC9-PSOD)	Network (0 = right temporal, 1 = background, 2 = frontal, 3 = visual, 4 = left temporal, 5 = motor)	Region (for labels please see Glasser52 labels table)	Degrees of freedom	T statistic	P value
**NETWORK ACTIVITY STRENGTH**	0	1		157	1.992	0
	0	4		157	-1.029	0.012
	1	1		157	1.52	0.021
	1	3		157	2.045	0.001
	1	4		157	-1.903	0.005
	2	3		157	2.355	0.001
**NETWORK ACTIVITY VARIABILITY**	0	1		157	2.181	0.003
	0	2		157	3.02	0
	0	3		157	1.921	0.009
	1	3		157	2.671	0.001
	2	3		157	2.575	0.004
	2	5		157	1.879	0.021
**COACTIVATION**	0	0		157	1.316	0.05
	0	5		157	1.813	0.008
	1	0		157	1.94	0.011
	1	1		157	-1.415	0.045
	1	2		157	1.81	0.016
	1	3		157	1.819	0.006
	1	4		157	1.96	0.01
	1	5		157	1.679	0.029
**DYNAMIC PARCEL COHERENCE**	0	0	0	157	1.313	0.023
	0	0	1	157	2.718	0.001
	0	0	2	157	1.468	0.011
	0	0	3	157	1.987	0.001
	0	0	7	157	1.281	0.026
	0	0	10	157	1.736	0.001
	0	0	13	157	1.257	0.032
	0	0	14	157	1.413	0.013
	0	0	15	157	2.079	0.001
	0	0	16	157	1.902	0.001
	0	0	17	157	1.781	0.001
	0	0	18	157	1.684	0.003
	0	0	19	157	2.959	0.001
	0	0	20	157	1.909	0.001

2

	0	0	22	157	1.652	0.004
	0	0	27	157	2.488	0.001
	0	0	28	157	1.58	0.006
	0	0	29	157	1.743	0.001
	0	0	32	157	1.65	0.004
	0	0	33	157	1.484	0.009
	0	0	40	157	1.384	0.014
	0	0	41	157	1.382	0.014
	0	0	42	157	1.239	0.033
	0	0	45	157	1.703	0.003
	0	0	46	157	1.702	0.003
	0	0	48	157	1.71	0.003
	0	0	49	157	1.385	0.014
	0	0	50	157	1.624	0.005
	0	0	51	157	1.241	0.033
	0	1	1	157	2.644	0.001
	0	1	3	157	1.689	0.003
	0	1	10	157	1.502	0.008
	0	1	15	157	1.836	0.001
	0	1	16	157	1.797	0.001
	0	1	17	157	1.627	0.005
	0	1	18	157	1.716	0.003
	0	1	19	157	2.804	0.001
	0	1	20	157	1.576	0.006
	0	1	21	157	1.24	0.033
	0	1	22	157	1.176	0.049
	0	1	27	157	2.451	0.001
	0	1	28	157	1.422	0.013
	0	1	29	157	1.722	0.003
	0	1	32	157	1.226	0.035
	0	1	33	157	1.409	0.014
	0	1	40	157	1.184	0.045
	0	1	42	157	1.398	0.014
	0	1	45	157	1.514	0.007
	0	1	46	157	1.619	0.005
	0	1	47	157	1.503	0.008
	0	1	49	157	1.207	0.043
	0	1	50	157	1.593	0.005
	0	2	1	157	2.684	0.001
	0	2	3	157	1.646	0.004
	0	2	15	157	1.569	0.007
	0	2	16	157	1.542	0.007

3

	0	2	17	157	1.281	0.026
	0	2	18	157	1.406	0.014
	0	2	19	157	2.586	0.001
	0	2	20	157	1.41	0.014
	0	2	27	157	2.32	0.001
	0	2	29	157	1.59	0.005
	0	2	45	157	1.274	0.026
	0	2	46	157	1.462	0.012
	0	3	0	157	1.345	0.019
	0	3	1	157	3.08	0.001
	0	3	2	157	1.606	0.005
	0	3	3	157	2.121	0.001
	0	3	7	157	1.339	0.021
	0	3	10	157	1.77	0.001
	0	3	14	157	1.582	0.006
	0	3	15	157	2.099	0.001
	0	3	16	157	2.201	0.001
	0	3	17	157	2.18	0.001
	0	3	18	157	1.874	0.001
	0	3	19	157	3.074	0.001
	0	3	20	157	1.963	0.001
	0	3	22	157	1.518	0.007
	0	3	27	157	2.941	0.001
	0	3	28	157	1.61	0.005
	0	3	29	157	1.804	0.001
	0	3	32	157	1.464	0.012
	0	3	33	157	1.889	0.001
	0	3	40	157	1.496	0.009
	0	3	41	157	1.402	0.014
	0	3	42	157	1.637	0.004
	0	3	45	157	1.737	0.001
	0	3	46	157	1.743	0.001
	0	3	47	157	1.417	0.013
	0	3	48	157	1.65	0.004
	0	3	49	157	1.474	0.01
	0	3	50	157	1.782	0.001
	0	3	51	157	1.233	0.033
	0	4	0	157	1.422	0.013
	0	4	1	157	2.947	0.001
	0	4	2	157	1.265	0.03
	0	4	3	157	2.047	0.001
	0	4	7	157	1.383	0.014

4

	0	4	10	157	1.644	0.004
	0	4	14	157	1.459	0.013
	0	4	15	157	2.016	0.001
	0	4	16	157	1.855	0.001
	0	4	17	157	1.506	0.007
	0	4	18	157	1.802	0.001
	0	4	19	157	3.008	0.001
	0	4	20	157	1.958	0.001
	0	4	21	157	1.443	0.013
	0	4	22	157	1.33	0.022
	0	4	27	157	2.599	0.001
	0	4	28	157	1.578	0.006
	0	4	29	157	1.878	0.001
	0	4	31	157	1.25	0.032
	0	4	32	157	1.45	0.013
	0	4	33	157	1.539	0.007
	0	4	40	157	1.558	0.007
	0	4	41	157	1.301	0.024
	0	4	42	157	1.556	0.007
	0	4	45	157	1.552	0.007
	0	4	46	157	1.663	0.003
	0	4	47	157	1.687	0.003
	0	4	48	157	1.34	0.021
	0	4	49	157	1.28	0.026
	0	4	50	157	1.478	0.01
	0	5	0	157	1.179	0.047
	0	5	1	157	3.008	0.001
	0	5	2	157	1.278	0.026
	0	5	3	157	1.879	0.001
	0	5	7	157	1.369	0.014
	0	5	10	157	1.357	0.017
	0	5	14	157	1.457	0.013
	0	5	15	157	2.055	0.001
	0	5	16	157	2.01	0.001
	0	5	17	157	1.993	0.001
	0	5	18	157	1.848	0.001
	0	5	19	157	3.111	0
	0	5	20	157	1.944	0.001
	0	5	22	157	1.357	0.018
	0	5	27	157	2.565	0.001
	0	5	28	157	1.539	0.007
	0	5	29	157	1.595	0.005

	0	5	32	157	1.3	0.024
	0	5	33	157	1.416	0.013
	0	5	40	157	1.405	0.014
	0	5	41	157	1.324	0.022
	0	5	42	157	1.207	0.043
	0	5	45	157	1.578	0.006
	0	5	46	157	1.492	0.009
	0	5	48	157	1.254	0.032
	0	5	50	157	1.533	0.007
	0	5	51	157	1.222	0.038
	1	0	19	157	1.817	0.022
	1	0	27	157	2.006	0.006
	1	0	29	157	1.673	0.029
	1	0	37	157	1.837	0.02
	1	1	19	157	1.942	0.009
	1	1	27	157	1.998	0.007
	1	1	28	157	1.586	0.035
	1	1	29	157	1.893	0.015
	1	1	37	157	1.965	0.007
	1	2	1	157	1.587	0.035
	1	2	3	157	1.576	0.037
	1	2	19	157	2.045	0.004
	1	2	27	157	1.967	0.007
	1	2	29	157	1.736	0.024
	1	2	37	157	1.832	0.02
	1	2	40	157	1.627	0.031
	1	3	3	157	1.524	0.048
	1	3	19	157	1.975	0.007
	1	3	27	157	1.905	0.012
	1	3	28	157	1.567	0.038
	1	3	29	157	1.751	0.024
	1	3	37	157	1.691	0.028
	1	4	3	157	1.537	0.044
	1	4	19	157	1.853	0.02
	1	4	27	157	1.796	0.022
	1	4	28	157	1.642	0.031
	1	4	29	157	2.064	0.002
	1	4	37	157	1.696	0.028
	1	4	40	157	1.616	0.032
	1	5	19	157	1.796	0.022
	1	5	27	157	1.774	0.023
	1	5	29	157	1.55	0.042

	1	5	37	157	1.692	0.028
	2	0	0	157	1.614	0.042
	2	0	1	157	2.124	0.006
	2	0	6	157	1.764	0.023
	2	0	27	157	1.625	0.039
	2	0	50	157	1.575	0.049
	2	1	0	157	1.668	0.032
	2	1	1	157	2.338	0.001
	2	1	6	157	1.778	0.021
	2	1	19	157	1.639	0.035
	2	1	27	157	1.889	0.015
	2	2	1	157	2.048	0.009
	2	3	1	157	2.012	0.009
	2	3	6	157	1.701	0.03
	2	3	27	157	1.638	0.036
	2	4	1	157	2.249	0.002
	2	4	6	157	1.941	0.013
	2	4	27	157	1.709	0.028
	2	5	1	157	2.189	0.005
	2	5	6	157	1.81	0.02
**DYNAMIC REGIONAL POWER**	0	0	39	157	3.624	0.026
